# Proteome and transcriptome analyses reveal key molecular differences between quality parameters of commercial-ripe and tree-ripe fig (*Ficus carica* L.)

**DOI:** 10.1186/s12870-019-1742-x

**Published:** 2019-04-16

**Authors:** Yuanyuan Cui, Ziran Wang, Shangwu Chen, Alexander Vainstein, Huiqin Ma

**Affiliations:** 1 Department of Fruit Tree Sciences, College of Horticulture, China Agricultural University, Beijing, 100193 China; 2 College of Food Science and Nutrition Engineering, China Agricultural University, Beijing, 100083 China; 3 Institute of Plant Sciences and Genetics in Agriculture, The Robert H. Smith Faculty of Agriculture, Food and Environment, The Hebrew University of Jerusalem, 76100 Rehovot, Israel

**Keywords:** *Ficus carica* L., Fruit, Commercial ripe, Tree ripe, Proteome, Transcriptome

## Abstract

**Background:**

Fig fruit are highly perishable at the tree-ripe (TR) stage. Commercial-ripe (CR) fruit, which are harvested before the TR stage for their postharvest transportability and shelf-life advantage, are inferior to TR fruit in size, color and sugar content. The succulent urn-shaped receptacle, serving as the protective structure and edible part of the fruit, determines fruit quality. Quantitative iTRAQ and RNA-Seq were performed to reveal the differential proteomic and transcriptomic traits of the receptacle at the two harvest stages.

**Results:**

We identified 1226 proteins, of which 84 differentially abundant proteins (DAPs) were recruited by criteria of abundance fold-change (FC) ≥1.3 and *p* < 0.05 in the TR/CR receptacle proteomic analysis. In addition, 2087 differentially expressed genes (DEGs) were screened by ≥2-fold expression change: 1274 were upregulated and 813 were downregulated in the TR vs. CR transcriptomic analysis. Ficin was the most abundant soluble protein in the fig receptacle. Sucrose synthase, sucrose-phosphate synthase and hexokinase were all actively upregulated at both the protein and transcriptional levels. Endoglucanase, expansin, beta-galactosidase, pectin esterase and aquaporins were upregulated from the CR to TR stage at the protein level. In hormonal synthesis and signaling pathways, high protein and transcriptional levels of aminocyclopropane-1-carboxylate oxidase were identified, together with a few diversely expressed ethylene-response factors, indicating the potential leading role of ethylene in the ripening process of fig receptacle, which has been recently reported as a non-climacteric tissue.

**Conclusions:**

We present the first delineation of intra- and inter-omic changes in the expression of specific proteins and genes of TR vs. CR fig receptacle, providing valuable candidates for further study of fruit-quality formation control and fig cultivar innovation to accommodate market demand.

**Electronic supplementary material:**

The online version of this article (10.1186/s12870-019-1742-x) contains supplementary material, which is available to authorized users.

## Background

Figs (*Ficus carica* L.), originating from Mediterranean regions, are among the earliest fruit consumed by man [1, 2]. The development of fig fruit can be divided into three stages; stage III, characterized by the fruit’s rapid increment in size, softening, color change and sugar accumulation, is the main phase of fruit-quality formation, and is much shorter than stages I or II, lasting only 3–10 days under different growing temperatures [3–5]. When the figs reach the more advanced ripening level—the tree-ripe (TR) stage, which can be regarded as complete physiological ripeness—the fruit are at their heaviest with highest soluble solids content, soft texture and best flavor. A consumer preference study demonstrated that most consumers are more willing to accept figs of TR quality than those of commercial-ripe (CR) quality [4]. However, due to the biological limitations of rapidly declining fruit texture, desiccation and phytopathogen infection [6], fresh figs are usually harvested at the CR stage, before they are fully ripe.

Structurally, the fig fruit is mainly composed of an inflorescence—flowers and receptacle. The receptacle forms the boundary of the flower fruitlets, the exocarp or pericarp of the fig in horticultural terms. As ripening proceeds, the skin color of the receptacle changes, providing an important source of anthocyanins [7–9]; sugars accumulate in the receptacle and the texture softens, constituting the edible part of the fig fruit. The receptacle is also a major tissue for latex production by laticiferous cells; latex has a protective function against insects and fungi [10], but a negative effect on fig taste.

Multi-omics provides a way of dealing with the complex and massive biological data of larger systems, enabling data integration and processing to gain insights into the interrelations, functioning and biological mechanisms at multiple levels of biological systems [11–15]. In recent years, several studies have been carried out on the transcriptomic aspects of fig fruit development and ripening. For example, the predicted transcriptomes of two major cultivars—Dottato and Horaishi—were compared, revealing more than 2000 and 4000 cultivar-specific genes, respectively [16]. Transcriptome comparison has also been applied to elucidate the expression differences of fig biotypes: a comparison between young San Pedro type fig and common fig fruit showed that zeatin biosynthesis and plant hormone signal-transduction pathways are differentially regulated [17], and ethylene synthesis and phytohormone signal transduction were found to be differentially expressed between caprifig and common fig fruit [18]. Fig fruit ripening and quality formation are of high interest for the industry. At the transcriptomic level, genes encoding ethylene-response factors (ERFs), fruit cell wall-modification enzymes and ascorbate oxidase were found to be strongly upregulated during fig ripening [19]. Transcriptomic evidence further revealed that the fig flesh and receptacle are of different climacteric natures during ripening, providing new insights for fig breeding and postharvest management [20].

The end product of gene expression, the protein, is the ultimate biofunction executor. Proteomic analysis may provide more direct information on key biological processes, such as fruit development and ripening [21]. Isobaric tags for relative and absolute quantitation (iTRAQ) are a widely used quantitative technology in proteomics studies [22, 23] that improves the accuracy and reliability of protein quantification over those based on the traditional 2D-polyacrylamide gel electrophoresis (PAGE) [24]. As such, it may provide a powerful tool to reveal and speculate on the complex and global changes underlying crucial fruit ripening, quality determination and horticultural trait formation, facilitating commercial and market-driven fig quality control and innovation.

As a perishable fruit, harvesting at different ripening stages has a great influence on postharvest maturation, senescence, quality formation and shelf life of fresh figs [3–5, 25]. However, the proteomic and transcriptomic profiles of fig at CR and TR stages are still largely unknown. In the present study, we compared the proteomes and transcriptomes of CR and TR fig receptacles to enhance our understanding of expression-profile and pathway changes during ripening of this historically and economically important fruit.

## Methods

### Plant materials

Common figs (*Ficus carica* var. Brown Turkey) were planted in the experimental station of China Agricultural University, Beijing, with of 3 × 3 m spacing. The fig trees were 5 years old, with standardized cultivation. CR fruit in the first half of stage III, and TR fruit in late stage III were collected in the summer of 2016. Each group consisted of 20 fruit, in triplicate, and samples were designated CR1, 2, 3 and TR1, 2, 3, respectively. Ten fruits from each biological replicate of each group were carried back to the laboratory for horticultural-attribute assays, and 10 fruits of each sample were cut into four sections with a scalpel in the field. After removing the female flowers, the receptacle tissues were quickly frozen in liquid nitrogen on site and then carried back to the laboratory and stored at − 80 °C for further analysis. A workflow chart of the whole process is demonstrated as Additional file 4: Figure S1.

### Horticultural attribute assays

Fig fruit size was measured with a vernier caliper, fruit weight with an electronic balance and fruit texture with a hardness tester (Fujiwara FHM-1, Japan). Soluble solids content was determined by refraction method using extruded juice. Starch content was determined with a starch content kit (Jiancheng A148, Nanjing, China) using ground powder, and acid content was determined by titration with 0.1 mol/L NaOH.

### RNA sequencing (RNA-Seq)

The thoroughly ground samples were subjected to RNA isolation, quality checking and mRNA enrichment as described in our previous publications [8, 17]. The cDNA libraries of the three biological replications of CR and TR fig samples were constructed with the Illumina Truseq™ RNA Sample Prep Kit according to the manufacturer’s instructions, then sequenced on the Illumina HiSeq platform [9, 17].

The original image data obtained by Illumina sequencing was transformed into sequence data by base calling, then the connector and low-quality sequences, as well as those that were too short or contained uncertain bases were filtered out to obtain high-quality sequence data. The final data were matched with our laboratory’s previous transcriptome database by RSEM software [17]. Gene expression was measured as fragments per kilobase of exon model per million mapped reads (FPKM). Differentially expressed genes (DEGs) were determined based on the negative binomial distribution by edgeR software according to gene read count data. The screening criteria for significant differences were: *p*-value (*p*-FDR) ≤ 0.05 & | log_2_(fold change) | ≥ 1. The RNA-Seq data have been deposited to the NCBI (SRA accession SRP135880) which was also used as local RNA-Seq assembly of fig receptacle gene expression dataset for auxiliary protein annotation.

### Protein extraction, digestion, labeling and mass spectrometry (MS)

The fig receptacle tissue samples were thoroughly ground to a very fine powder in liquid nitrogen, and soluble proteins were extracted using the trichloroacetic acid (TCA)–ice-cold acetone precipitation method (1.0 g sample powder was combined with 4 mL of 10% *w*/*v* TCA in acetone) as described in our previous publication [26]. Protein concentration was measured by the Bradford method, and 10 μg protein from each sample was separated by sodium dodecyl sulfate (SDS)-PAGE. The whole gel for each sample was cut into eight continuous sections, which were then respectively placed in centrifuge tubes, digested for 24 h at 37 °C after decolorization (1 μg/μL trypsin stock solution (Promega, Madison, WI, USA) diluted 15 times in 25 mM triethylamine borane (TEAB), with 3.3 μg of trypsin for 100 μg protein. The digested peptides were lyophilized and then reconstituted in 50% (*w*/*v*) TEAB. Six samples were labeled with iTRAQ Reagent-8PLEX Multiplex Kit (ABSciex Inc., MA, USA) according to the manufacturer’s instructions. CR1, CR2 and CR3, TR1, TR2 and TR3 were labeled with iTRAQ reagents of 115, 116 and 117, 118, 119 and 121 Da, respectively. All labeled samples were lyophilized, then redissolved in Milli-Q water and desalted using a Strata-X-C cartridge (Phenomenex, Torrance, CA, USA).

The protein samples were dissolved in 50 μL of mobile phase A (H_2_O, 0.1% *w*/*v* formic acid) and loaded onto an Acclaim PePmap C18-reversed phase liquid chromatography (LC) column (75 μm × 2 cm, 3 μm, 100 Å, Thermo Fisher Scientific, Waltham, MA, USA), and then separated with a reversed-phase C18 column (75 μm × 10 cm, 5 μm, 300 Å, Agela Technologies, USA) mounted on a Dionex ultimate 3000 nano LC system. Peptides were eluted at a flow rate of 400 nL/min using a gradient of 5–80% (*v*/v) acetonitrile in 0.1% formic acid. The eluates were injected directly into a Q-Exactive mass spectrometer (Thermo Fisher Scientific) set in positive ion mode and a data-dependent manner with full MS scan 350–2000 m/z, full scan resolution at 70,000, MS/MS scan resolution at 17,500, MS/MS scan with minimum signal threshold 1E+ 5, isolation width at 2 Da. To evaluate the performance of this MS on the iTRAQ-labeled samples, two MS/MS acquisition modes and higher-energy collisional dissociation (HCD) were employed. To optimize the MS/MS acquisition efficiency of HCD, normalized collision energy (NCE) was systemically examined 28%.

The MS results were input into Proteome Discoverer 1.3, with the following parameters: master ion mass range of 350–6000 Da; minimum number of peaks in the second mass spectrum, 10; signal-to-noise ratio (SNR) S/N threshold of 1.5. The identification and quantitation of the selected peptides were performed by Mascot search engine (version 2.3.01 Matrix Science, London) against uni_Moraceae_3487 (http://www.uniprot.org/taxonomy/3487) and our local RNA-Seq assembly dataset (SRA accession is SRP135880), the false-positive rate (FDR) was controlled at < 1%. The user-defined search parameters were fixed modification: carbamidomethyl (C); variable modification: oxidation (M), Gln → Pyro-Glu (N-term Q), iTRAQ 8PLEX (K), iTRAQ 8PLEX (Y), iTRAQ 8PLEX (N-term); peptide tol: 15 ppm; MS/MS tol: 20 mmu; max missed cleavages: 1. Quantitative analysis parameters were protein ratio type: median; minimum peptides: 1; normalization method median: 1. Differentially abundant proteins (DAPs) were determined based on iTRAQ-2DLC-MS/MS triplicate peak area measurements (115, 116, 117 Da for CR, and 118, 119, 121 Da for TR), and the averaged prior-to-calculating ratios (Additional file 5: Figure S2). iTRAQ ratios below the low range (≤0.77) or above the high range (≥1.3) (arbitrary fold changes (FC) ≥1.3) were considered to be downregulated or upregulated, respectively, with fold change *p*-value (*p*-FDR) ≤ 0.05 according to [27, 28]. Gene ontology (GO)-enrichment analysis was conducted by Goatools with Fisher’s precise test, the *p*-values were corrected by multiple testing to control the FDR. GO enrichment was considered significant when the corrected *p*-value (*p*-FDR) ≤ 0.05. Kyoto Encyclopedia of Genes and Genomes (KEGG)-pathway enrichment analysis was performed by KOBAS (http://kobas.cbi.pku.edu.cn/index.php) with Fisher’s precise test and the significance analysis was the same as for GO enrichment. The mass spectrometry proteomics data have been deposited to the ProteomeXchange Consortium (www.proteomexchange.org) [29] via the PRIDE partner repository with the dataset identifier PXD009757.

### Verification by real-time quantitative PCR

Total RNA of each biological replication was reverse transcribed with PrimeScript RT Reagent Kit with gDNA Eraser (Takara, Dalian, China) according to the manufacturer’s instructions, and concentrations were adjusted to < 100 ng/μL, as measured in a Nanodrop 2000 spectrophotometer. The gene-specific primer pairs were designed online (http://bioinfo.ut.ee/primer3/) (Additional file 1: Table S1), and cDNA content was normalized to *actin* as described previously [17]. Reactions were performed with SYBR Premix Ex Taq II in an ABI 7500 real-time PCR system. Reaction mixture (20 μL) was added to each well. The initial denaturation was set at 95 °C for 30 s and the thermal cycle was set at 95 °C for 5 min, 60 °C for 34 s, 40 cycles in total. The output results were analyzed by Excel 2010. The real-time PCR was conducted with three replicates for each sample.

## Results

### Horticultural differences between CR and TR figs

**Fig. 1:**
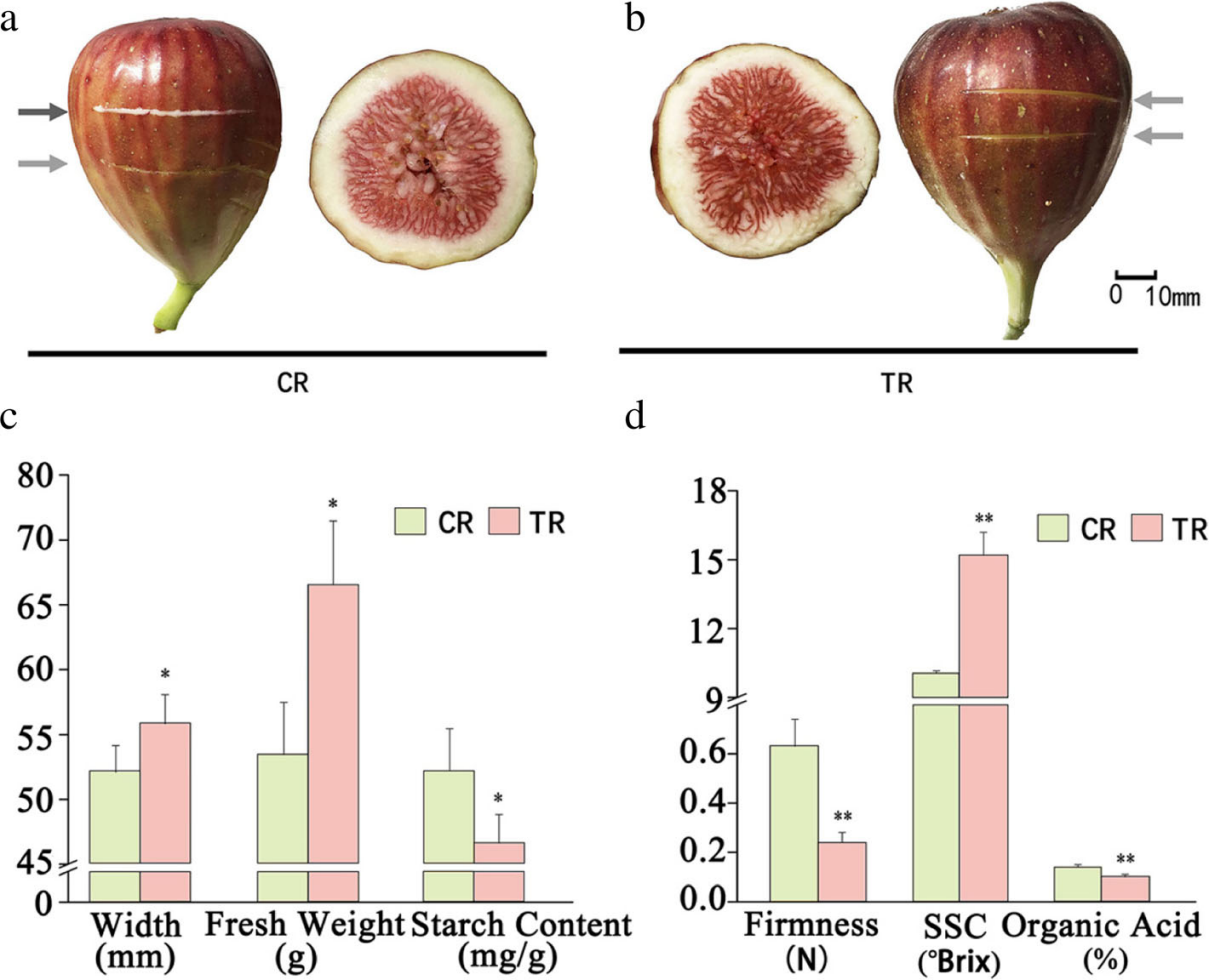
Fruit and horticultural characteristics of tree-ripe (TR) and commercial-ripe (CR) Brown Turkey figs. **a** CR fig and transection of the fruit. **b** TR figs and transection of the fruit. Arrows indicate fruit peel abrasions with (dark arrow) and without (light arrows) latex. **c** Transection diameter (width), fruit fresh weight and starch content for CR and TR figs. **d** Fruit firmness, soluble solids content in °Brix and titratable organic acid of CR and TR figs. *, **Significantly different at *p* ≤ 0.05 and 0.01, respectively

‘Brown Turkey’ CR fig was slightly softened and colored red with a yellow-green background, while the area near the neck was less colored. Apparent latex flow was observed when the fruit were gently cut with a scalpel; the receptacle tissue was thick, with a firm appearance and a clear boundary, with pink-colored female flower tissues. TR fig had a deeper reddish color and darker hues; there was no latex when the fruit was cut with a scalpel (Fig. 1a, b). The TR fruit was soft, with 61.9% less firmness compared to CR, while fruit size expanded by about 7% in average transverse diameter, and the average fresh weight increased by 24%. The soluble solids content showed a 33% increment TR vs. CR fruit, accompanied by a 10% decrease in starch content, and a decrease in organic acid from 0.14 to 0.10% (Fig. 1c, d).

### Proteomic and transcriptomic characteristics of the ripening fig receptacle

**Fig. 2:**
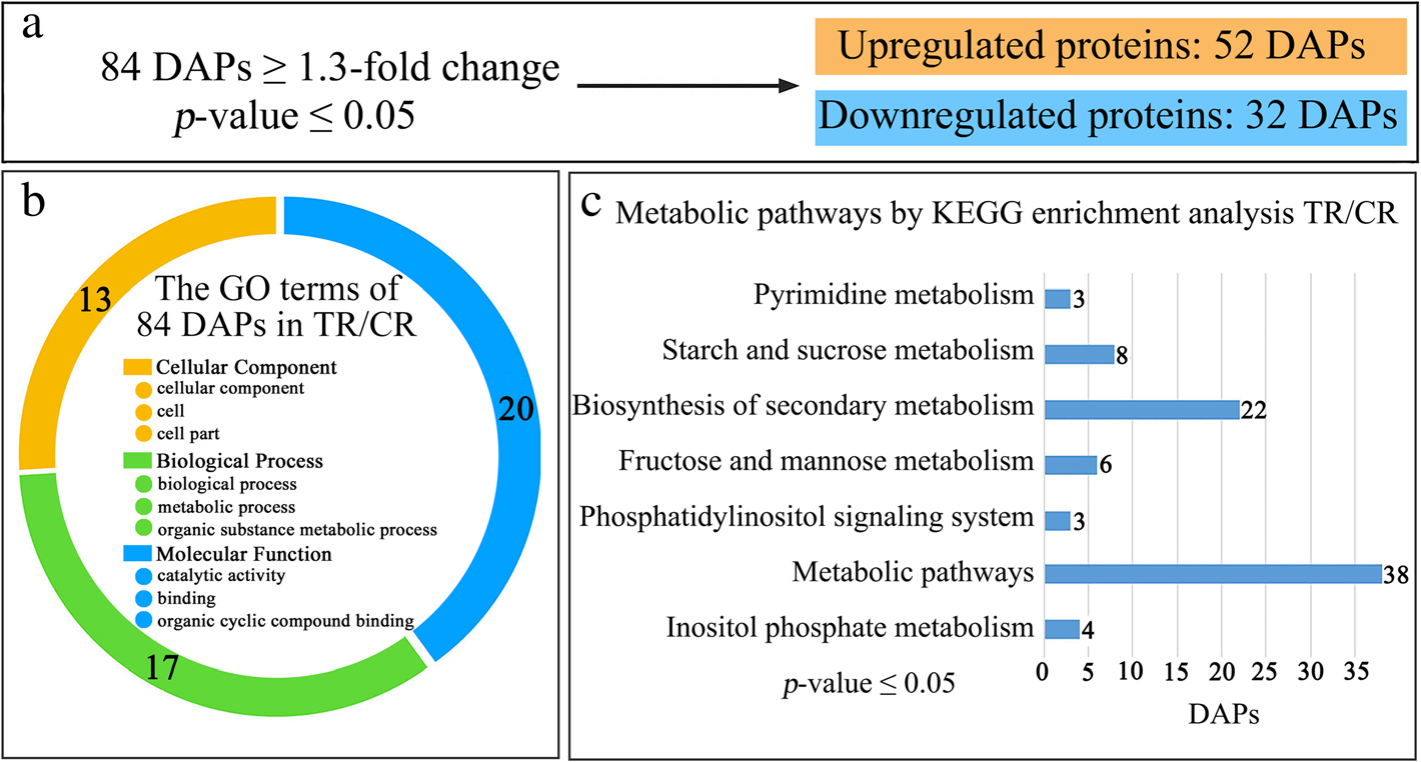
iTRAQ proteomic profiles of differentially abundant proteins (DAPs) annotated in Moraceae and RNA-Seq assembly of tree-ripe (TR) and commercial-ripe (CR) Brown Turkey figs. **a** Number of upregulated and downregulated DAPs recruited by fold-change ≥1.3 and *p* ≤ 0.05. **b** GO term attribution for DAPs. **c** KEGG pathway enrichment for DAPs

iTRAQ proteomic and RNA-Seq transcriptomic analyses were performed on the CR and TR fig receptacle tissues (Additional file 7: Figure S1). A total of 1226 proteins were annotated against uni_Moraceae_3487 and our local fig RNA-Seq assembly dataset (Additional file 3: Table S3), and 84 of them were screened as DAPs (with FC ≥ 1.3, *p* ≤ 0.05), with 52 upregulations and 32 downregulations in TR vs. CR figs (Fig. 2a). The DAPs’ GO terms were mostly assigned to Molecular Function and Biological Process, with 20 and 17 GO terms, respectively; 13 were assigned to Cellular Component (Fig. 2b). In Molecular Function, they focused mainly on catalytic activity; In Biological Process, GO terms of the DAPs were mainly attributed to metabolic process. Most of DAPs were involved in metabolic pathways and biosynthesis of secondary metabolites according to KEGG analysis with *p* ≤ 0.05. Thirty-eight DAPs were enriched in metabolic pathways and 22 were enriched in biosynthesis of secondary metabolites (Fig. 2c). DAPs related to hormonal synthesis and signaling pathways enclosed in biological process were mapped.

**Fig. 3:**
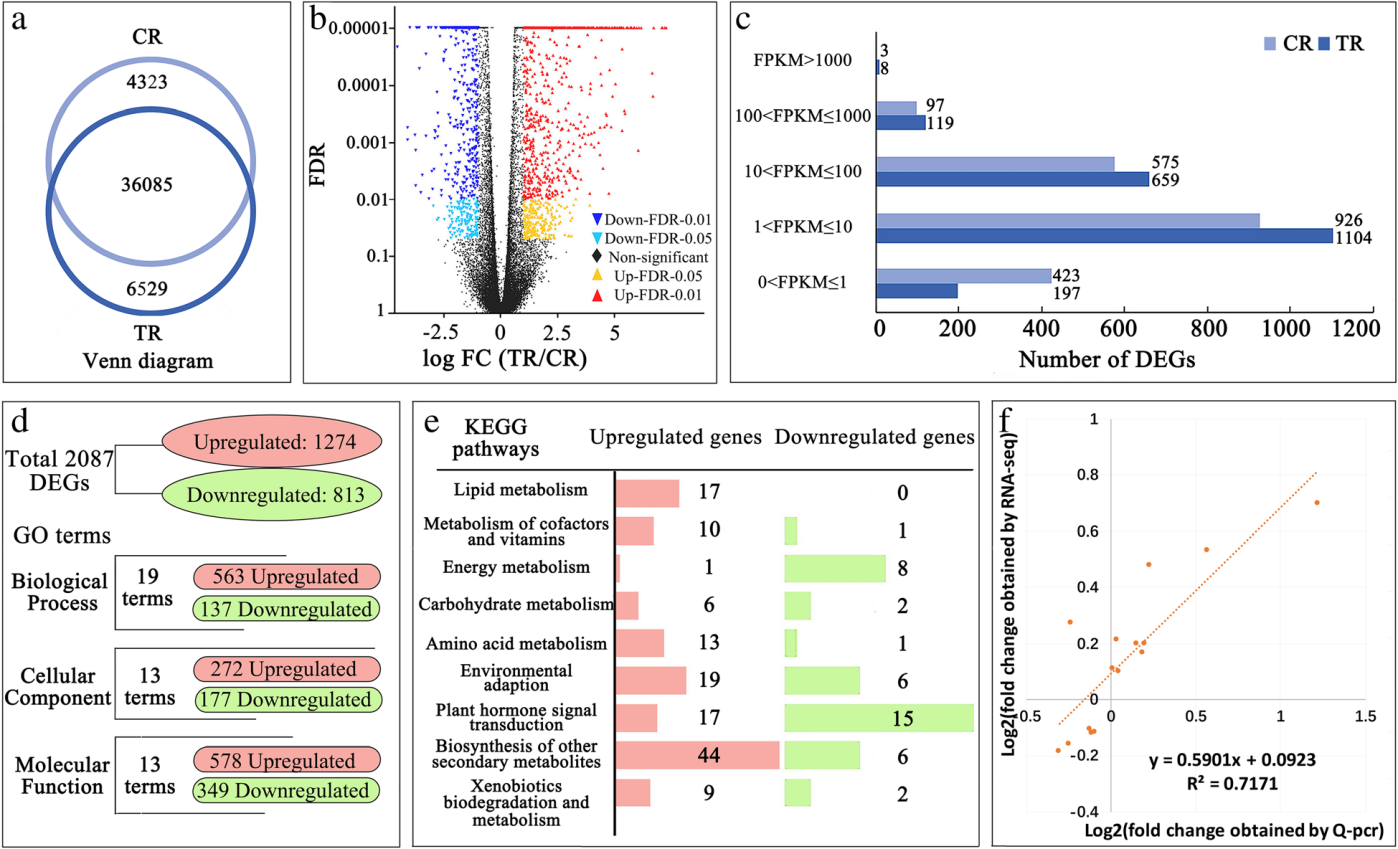
The general profiles of RNA-Seq transcriptomes of tree-ripe (TR) and commercial-ripe (CR) Brown Turkey figs. **a** Venn diagram of the total mapped genes in triplicate CR and TR receptacle samples. **b** Volcano diagram of the distribution of CR and TR gene sets. More upregulated genes (red dots) were found than downregulated genes (blue dots). **c** Transcription frequency and abundance of differentially expressed gene (DEG) groups displayed as FPKM values in CR and TR. The number of genes in a group is listed to the right of the bar. **d** Outline of total DEGs, and analysis of GO term distribution for 2087 DEGs in Biological Process, Cellular Component and Molecular Function GO categories. Number of upregulated and downregulated DEGs is highlighted in red and green, respectively. **e** KEGG pathway enrichment analysis of the DEGs. Upregulated and downregulated DEGs are indicated by red and green bars, respectively, and to the right of each bar is the number of DEGs. **f** RNA-Seq validation and correlation analysis by real-time quantitative PCR

The RNA-Seq transcriptome of CR and TR receptacles yielded a total of 46,937 genes. A Venn diagram showed that 36,085 of these genes are expressed in both CR and TR receptacles, 4323 are expressed specifically in CR, and 6529 are expressed specifically in TR. (Fig. 3a); 2087 DEGs were screened between the TR and CR samples: 1274 were upregulated and 813 were downregulated in TR vs. CR fig receptacles; thus, the number of upregulated genes was remarkably higher than the number of downregulated genes (Fig. 3b). Expression of about 5% of the DEGs was high (FPKM ~ 100–1000) and very high (FPKM > 1000), and about 70% of them had expression of ~ 1–100 FPKM (Fig. 3c). Further GO term annotation in the three major GO categories indicated that Biological Process had more DEGs than Cellular Component or Molecular Function (Fig. 3d). KEGG analysis of the total DEGs showed enrichment in nine significant pathways. The pathways showing the largest changes were secondary metabolite biosynthesis (including flavonoid biosynthesis, phenylpropanoid biosynthesis), plant hormone-signal transduction, environmental adaptation, and lipid and energy metabolism. Upregulated genes were mainly assigned to biosynthesis of other secondary metabolites; downregulated genes were mainly assigned to plant hormone signal transduction (Fig. 3e).

Real-time quantitative PCR was applied to a set of 15 genes with different FPKM values in CR and TR figs. Linear regression analysis revealed a high correlation coefficient (R^2^ = 0.717) between the transcriptome and real-time PCR results, validating the RNA-Seq results (Fig. 3f, Additional file 6: Figure S3).

### Latex-related proteins, protein synthesis and turnover

**Fig. 4:**
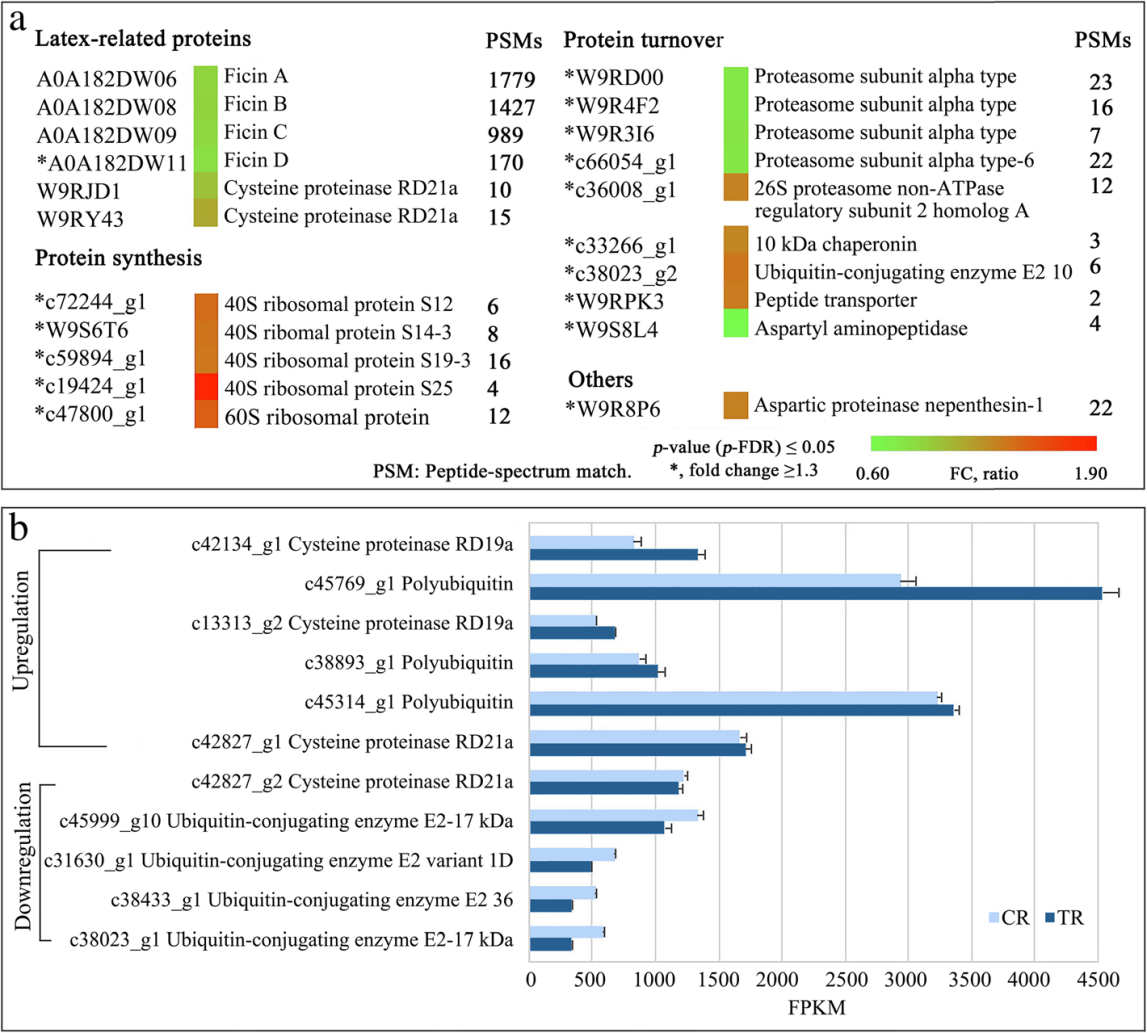
Latex- and protein turnover-related proteins and genes in tree-ripe (TR) and commercial-ripe (CR) Brown Turkey figs. **a** Heat map of differentially abundant proteins (DAPs). The ficins and cysteine proteinase sequences were aligned (Additional file 7: Figure S4); number of peptide spectrum matches (PSMs) indicates the relative abundance of the DAPs. **b** High-FPKM differentially expressed genes (FPKM > 500 in at least one of the pairs) related to proteinases and protein turnover of triplicate CR and TR samples

Ficin is the major protein identified in fig latex [30]. In the present study, it was identified as the most abundant soluble protein in the receptacle tissues of both CR and TR figs with high sequence similarity (Additional file 7: Figure S4). Specifically, ficin C and B were the main isoforms, followed by ficin A, while ficin D was the least abundant isoform (Additional file 3: Table S3). Ficin A, C, B and D decreased 0.83-, 0.84-, 0.81- and 0.77-fold, respectively, in TR fig compared to CR fig, whereas only ficin D abundance change reached the preset DAPs significant difference criteria (Fig. 4a). Two cysteine proteinase RD21a proteins (Uniprot Accession: W9RJD1, W9RY43) were identified with 66.6% amino acid sequence similarity, of which the center sections shared strong similarities with ficin (Additional file 7: Figure S4), although their abundance did not differ between the two ripening levels (Fig. 4a). At the transcriptional level, the RNA coding for ficin isoforms was not annotated. C*ysteine proteinase RD19a*, *RD21a* and *thiol protease aleurain* were identified, all belong to the papain family cysteine proteases with FPKM of ~ 500–1600, and no significant difference in expression between CR and TR figs (Fig. 4b).

For the protein-synthesis and turnover, significant increment was found with 4 40S and 1 60S ribosomal proteins (Fig. 4a). Correspondingly, 30 DEGs were annotated as ribosomal proteins, 12 encoding 40S and 18 60S, and these were upregulated in the TR receptacle (Additional file 2: Table S2). DAPs related protein-turnover were mainly upregulated, including 26S proteasome, chaperonin, ubiquitin-protein ligase, ubiquitin-conjugating enzyme and peptide transporter, while proteasomes demonstrated significant downregulation in TR vs. CR fruit (Fig. 4a). It is interesting to note that the aspartic proteinase nepenthesin-1 was upregulated in TR fig (Fig. 4a). At present, there is only a limited understanding of the production and induction of aspartic proteinase in *Nepenthes* plants. However, some studies have shown that a series of protease changes following predation by *Nepenthes* are similar to those in interactions between plants and pathogens or pests [31].

At the mRNA level, a large number of genes related to ubiquitination were identified, and genes with FPKM ≥500 were mainly annotated as *ubiquitin-conjugating enzyme E2* and *polyubiquitin*; the former showed a downward trend, and the latter an upward trend with ripening, but neither change was significant (Fig. 4b). Twenty DEGs were annotated as encoding proteinases, including subtilisin-like and serine protease, aspartic proteinase nepenthesins, and metalloendoproteinases: 16 of these were downregulated and 4 upregulated in TR vs. CR fruit. Eight DEGs were identified as encoding proteinase inhibitors, most of them subtilisin inhibitors. Of the DEGs encoding ubiquitin-related proteins, 15 were upregulated, 3 were downregulated, and both *proteasome core complex protein* and *26S proteasome non-ATPase regulatory* DEGs were upregulated in TR vs. CR fruit (Additional file 2: Table S2).

### Sugar accumulation

Key enzymes in sucrose accumulation were identified; sucrose synthase 2 was upregulated 1.28-fold in TR vs. CR figs (Table 1). Blasting of the protein sequence revealed that the upregulated sucrose synthase had high similarity to *Vitis vinifera* sucrose synthase (88.7%) and *Citrus unshiu* sucrose synthase (84.4%) (Additional file 8: Figure S5). One sucrose-phosphate synthase (SPS) was identified, with 1.52-fold change between ripening stages; this protein showed 87.3% similarity to SPS 1f of *Prunus persica* and 86.7% similarity to SPS of *Citrus unshiu* (Table 1, Additional file 8: Figure S5). Besides, hexokinase-1 was also upregulated 1.36-fold in TR vs. CR figs (Table 1).

**Table 1:** Key and differentially expressed proteins in sugar accumulation

Accession	Description	Annotation database	*p*-value	Ratio	PSMs
c26558_g1	Sucrose synthase 2	RNA-Seq assembly	0.00	1.28	35
^a^c44769_g2	Sucrose-phosphate synthase 1	RNA-Seq assembly	0.03	1.52	10
^a^c41752_g1	Hexokinase-1	RNA-Seq assembly	0.04	1.36	12
^a^W9S032	Alpha-1,4 glucan phosphorylase	*Morus notabilis*	0.00	1.34	25
^a^W9QQH1	Fructose-bisphosphate aldolase	*Morus notabilis*	0.00	0.75	66
^a^W9S728	Triosephosphate isomerase	*Morus notabilis*	0.00	0.76	45
^a^W9R6S4	Triosephosphate isomerase	*Morus notabilis*	0.00	0.61	10
^a^W9S4U6	Triosephosphate isomerase	*Morus notabilis*	0.01	0.66	18
^a^W9SM44	GDP-mannose 3,5-epimerase 1	*Morus notabilis*	0.01	0.73	4
^a^c43883_g3	UDP-glucose glucosyltransferase	RNA-Seq assembly	0.00	1.31	122

^a^fold change ≥1.3; PSM, peptide-spectrum match; Ratio: tree-ripe/commercial-ripe

Alpha-1,4 glucan phosphorylase and glycosyltransferase, related to starch and amylose metabolism, were mainly upregulated in TR vs. CR fruit. On the other hand, enzymes related to fructose and glucose metabolism were mainly downregulated, including fructose-bisphosphate aldolase and triosephosphate isomerase (Table 1).

**Fig. 5:**
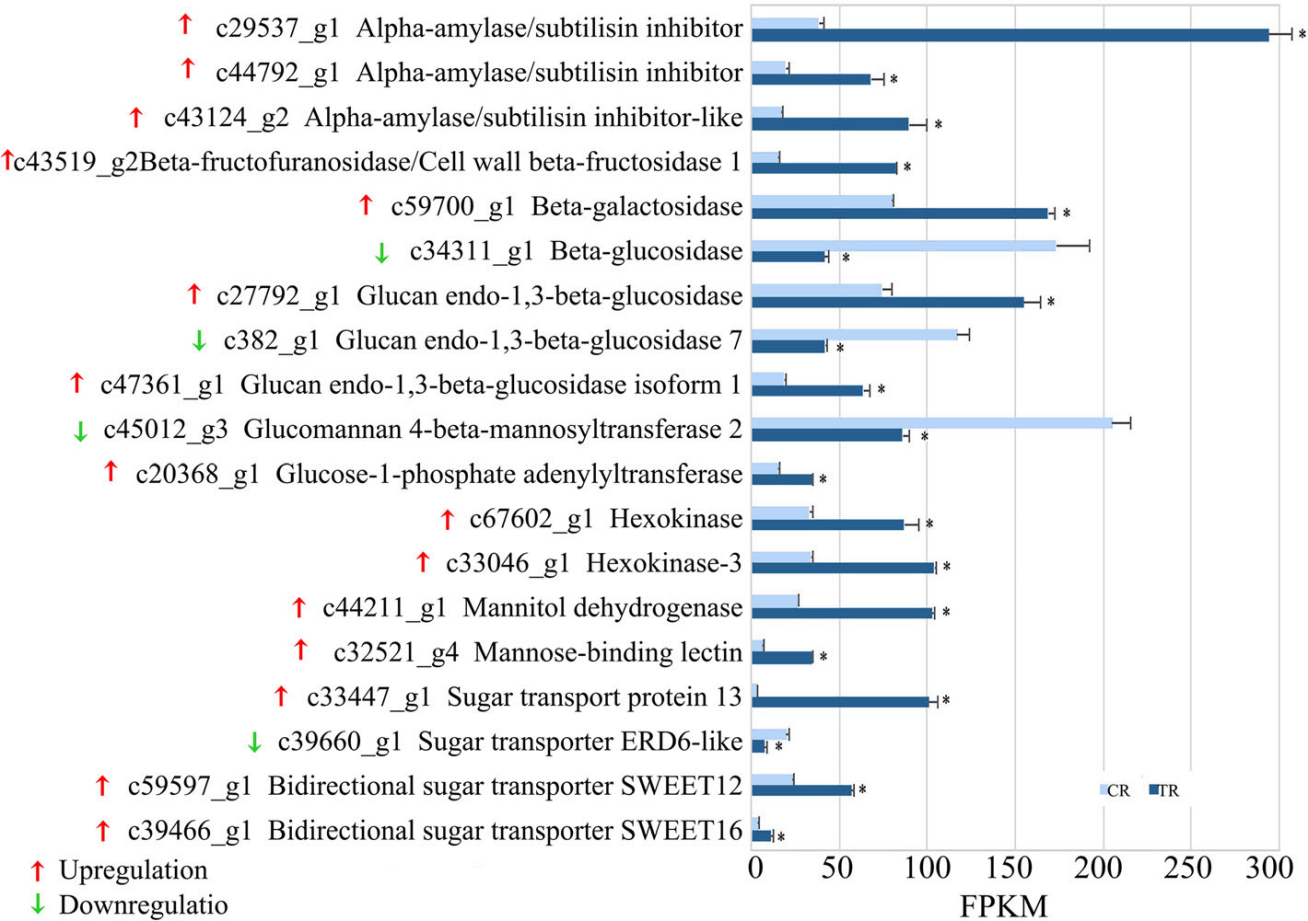
Sugar accumulation-related differentially expressed genes of tree-ripe (TR) and commercial-ripe (CR) Brown Turkey figs. *Significantly different at *p*-value (*p*-FDR) ≤ 0.05 and| log_2_FC | > = 1

DEGs related to sugar accumulation were mainly upregulated in TR vs. CR fruit (Fig. 5). The three alpha-amylase genes (*c29537_g1, c44792_g1, c43124_g2*) were upregulated in TR figs. *Beta-fructofuranosidase*, *beta-galactosidase, glucose-1-phosphate adenylyltransferase, hexokinase, mannitol dehydrogenase* and *mannose-binding lectin* were all upregulated; for *beta-fructofuranosidase* and *mannose-binding lectin*, the difference in expression was over 5-fold (Fig. 5). *Beta-glucosidase* and *glucomannan 4-beta-mannosyltransferase 2* expression was downregulated, while three genes encoding glucan endo-1,3-beta-glucosidases showed divergent changes in expression (Fig. 5). Sucrose synthase-encoding genes were not significantly differentially expressed at the RNA level between CR and TR figs (Additional file 9: Figure S6).

Among the sugar transporters, the largest expression increase in terms of fold change was found with *sugar transport protein 13*, whose expression in TR figs was nearly 33-fold that in CR figs. Furthermore, *bidirectional sugar transporter SWEET12* was upregulated 2.43 times in the TR fig receptacle (Fig. 5).

In addition, we identified three highly expressed raffinose synthase-encoding genes, although their FPKM values were almost unchanged between samples. Four high-FPKM sugar transporter-encoding genes with no significant difference in expression between CR and TR figs were also identified; among them, the FPKM of *bidirectional sugar transporter N3* increased from 818.18 in CR figs to 1266.33 in TR figs (Additional file 9: Figure S6).

### Proteins related to modifications in fruit firmness and texture

DAPs related to modifications in cytoskeleton, cell wall and cell connections, and to osmotic pressure regulation were identified in the two fig samples. A relatively highly expressed leucine-rich repeat extensin-like protein was significantly downregulated in TR vs. CR fruit, together with a pectate lyase and a glucan endo-1,3-beta-glucosidase present in relatively low amounts. Upregulation in TR fruit was found for three endoglucanases, expansion, pectate lyase 20, two beta-galactosidases and one pectinesterase. Endoglucanases, expansion and pectate lyase were up to 1.3-fold change. Water is an important component of cell turgor pressure. Aquaporin PIP2–1, aquaporin PIP2–2 and aquaporin PIP2–5 belonging to plasma membrane intrinsic proteins were upregulated 1.72-, 1.50- and 1.48-fold, respectively, in TR vs. CR figs. Pyrophosphate-energized vacuolar membrane proton pump was also upregulated 1.3-fold. The increase in aquaporin and vacuolar membrane proton pump contents was positively related to the size and weight increments of the TR fruit (Table 2).

**Table 2:** Important and differentially expressed proteins involved in fruit firmness and texture

Accession	Description	Annotation database	*p*-value	Ratio	PSMs
Cell wall and extracellular modification					
^a^c44496_g1	Endoglucanase	RNA-Seq assembly	0.02	1.85	10
^a^c29668_g1	Endoglucanase 24	RNA-Seq assembly	0.00	1.48	56
^a^Q6RX19	Endoglucanase 1 (Fragment)	*Morus notabilis*	0.00	1.54	9
^a^c25741_g1	Expansin-A11	RNA-Seq assembly	0.00	1.63	49
^a^c33895_g4	Pectate lyase 20	RNA-Seq assembly	0.04	1.31	45
W9QGU8	Leucine-rich repeat extensin-like protein 4	*Morus notabilis*	0.00	0.83	49
W9R5Y9	Beta-galactosidase	*Morus notabilis*	0.00	1.20	16
W9QT05	Beta-galactosidase 10	*Morus notabilis*	0.00	1.25	2
W9RQH1	Pectinesterase	*Morus notabilis*	0.00	1.06	15
W9S1B9	Pectate lyase	*Morus notabilis*	0.00	0.81	4
W9RMX9	Glucan endo-1,3-beta-glucosidase	*Morus notabilis*	0.00	0.77	3
Osmotic pressure related					
^a^c24882_g1	Aquaporin PIP2–1	RNA-Seq assembly	0.01	1.72	4
^a^W9S557	Aquaporin PIP2–2	*Morus notabilis*	0.01	1.50	5
^a^c40402_g2	Aquaporin PIP2–5	RNA-Seq assembly	0.00	1.48	5
^a^c46349_g1	Pyrophosphate-energized vacuolar membrane proton pump	RNA-Seq assembly	0.00	1.30	8

^a^fold change ≥1.3; Ratio, tree-ripe/commercial-ripe; *PSM* Peptide-spectrum match

**Fig. 6:**
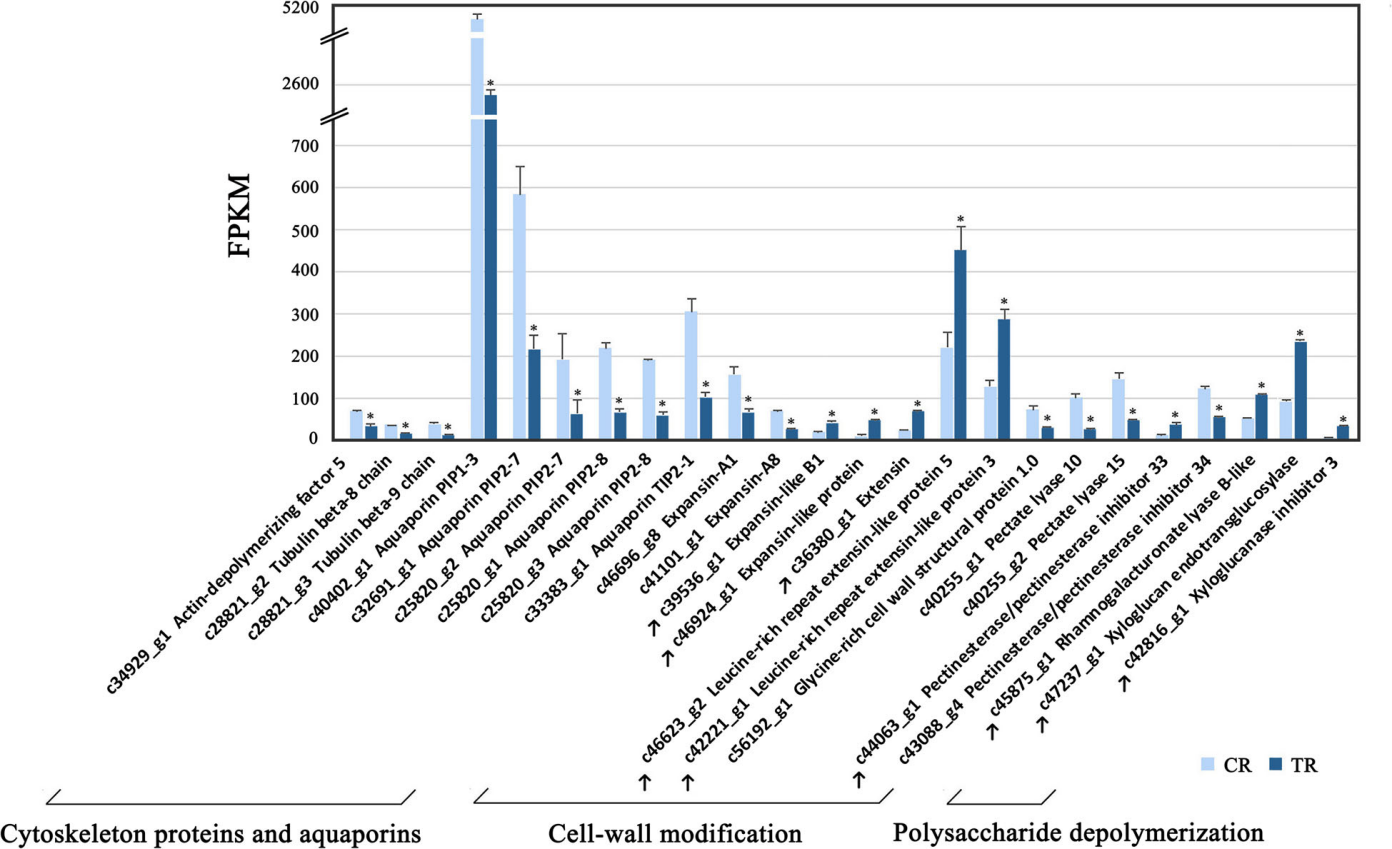
Cell expansion-related differentially expressed genes of tree-ripe (TR) and commercial-ripe (CR) Brown Turkey figs. *Significantly different at *p*-value (*p*-FDR) ≤ 0.05 and | log_2_FC | > = 1; ↑ indicates upregulation

DEGs associated with pectin degradation and cell-wall modification were mainly downregulated in TR vs. CR fruit, including: *pectate lyase*, *pectinesterase/pectinesterase inhibitor*, *expansin*, and some genes related to cell-wall-polysaccharide depolymerization (Fig. 6). *Pectate lyase*s *10* and *15* were downregulated; *pectinesterases (pectinesterase/pectinesterase inhibitor 33*, *34)* and *expansins (expansin-A1, expansin-A8, expansin-like B1, expansin-like protein, extension, leucine-rich repeat extensin-like protein 3, leucine-rich repeat extensin-like protein 5*) showed divergent expression. Genes related to cell-wall-polysaccharide depolymerization were all upregulated in TR vs. CR fruit, including *rhamnogalacturonate lyase B-like*, *xyloglucan endotransglucosylase* and *xyloglucanase inhibitor 3*. Moreover, *glycine-rich cell wall structural protein 1.0* was identified as downregulated in TR fruit to 0.43 its expression level in CR fruit (Fig. 6). Aside from genes involved in apoplast modification, downregulated expression was found for cytoskeleton protein-encoding genes: *actin-depolymerizing factor 5, tubulin beta-8 chain* and *tubulin beta-9 chain*, in TR fruit. Interestingly, a large number of high-FPKM value aquaporin genes were downregulated, of which *aquaporin PIP1–3*, with the highest FPKM, decreased from 5004.28 to 2413.2 (Fig. 6).

### Ethylene synthesis, plant hormones and secondary metabolites

The two key enzymes in the ethylene-synthesis pathway—ACO (A0A0G3FFL2) and s-adenosylmethionine synthase—decreased in TR figs to 0.7 of their levels in CR figs, while ACO was still found to be highly abundant in fig receptacle tissues during ripening (Table 3).

**Table 3:** Differentially expressed proteins in ethylene synthesis, secondary metabolites, oxidoreductase activity and stress response

Accession	Description	Annotation database	*p*-value	Ratio	PSMs
Ethylene					
^a^A0A0G3FFL2	1-Aminocyclopropane-1-carboxylate oxidase	*Morus notabilis*	0.00	0.70	113
^a^W9QYE6	S-adenosylmethionine synthase	*Morus notabilis*	0.01	0.70	15
Secondary metabolites					
^a^c78174_g2	Anthocyanidin 3-O-glucosyltransferase	RNA-Seq assembly	0.03	1.42	45
^a^G3F8M2	CHS1 (fragment)	*Morus notabilis*	0.00	1.39	4
^a^c41516_g1	2-C-methyl-D-erythritol 2,4-cyclodiphosphate synthase	RNA-Seq assembly	0.01	1.30	3
Oxidoreductase activity					
^a^c53614_g1	L-ascorbate oxidase homolog	RNA-Seq assembly	0.01	1.55	59
^a^c59788_g1	Monodehydroascorbate reductase	RNA-Seq assembly	0.00	1.31	39
^a^W9QH65	Glutathione peroxidase	*Morus notabilis*	0.00	1.45	4
^a^Q8S3U4	Peroxidase	*Morus notabilis*	0.00	0.73	39
^a^W9SM58	Peroxidase	*Morus notabilis*	0.00	0.76	22
^a^W9RAV1	Glutamate decarboxylase	*Morus notabilis*	0.02	0.77	8
^a^c46417_g1	Glutathione S-transferase DHAR2	RNA-Seq assembly	0.02	0.76	10
^a^c45114_g1	Glutathione S-transferase	RNA-Seq assembly	0.01	0.64	8
^a^c34584_g1	Glutathione S-transferase	RNA-Seq assembly	0.02	0.70	19
^a^c27863_g2	Lactoylglutathione lyase	RNA-Seq assembly	0.00	0.76	5
^a^W9S0A5	2-Cys peroxiredoxin BAS1-like protein	*Morus notabilis*	0.01	0.77	3
^a^c43305_g1	D-3-phosphoglycerate dehydrogenase	RNA-Seq assembly	0.00	0.75	26
Stress response					
^a^c41026_g2	Major allergen Bet v 1 homolog	RNA-Seq assembly	0.01	1.50	6
^a^c30948_g1	Major allergen Pru av. 1 homolog	RNA-Seq assembly	0.00	3.15	137
^a^c78934_g1	Minor allergen Alt a 7 homolog	RNA-Seq assembly	0.00	0.75	2
^a^c47341_g1	Acidic endochitinase	RNA-Seq assembly	0.00	1.83	24
^a^c39769_g1	Dehydrin COR47	RNA-Seq assembly	0.00	1.84	24
^a^c47235_g2	Pleiotropic drug resistance protein 3	RNA-Seq assembly	0.00	1.49	18

^a^fold change ≥1.3; ratio, tree-ripe/commercial-ripe; *PSM* Peptide-spectrum match

**Fig. 7:**
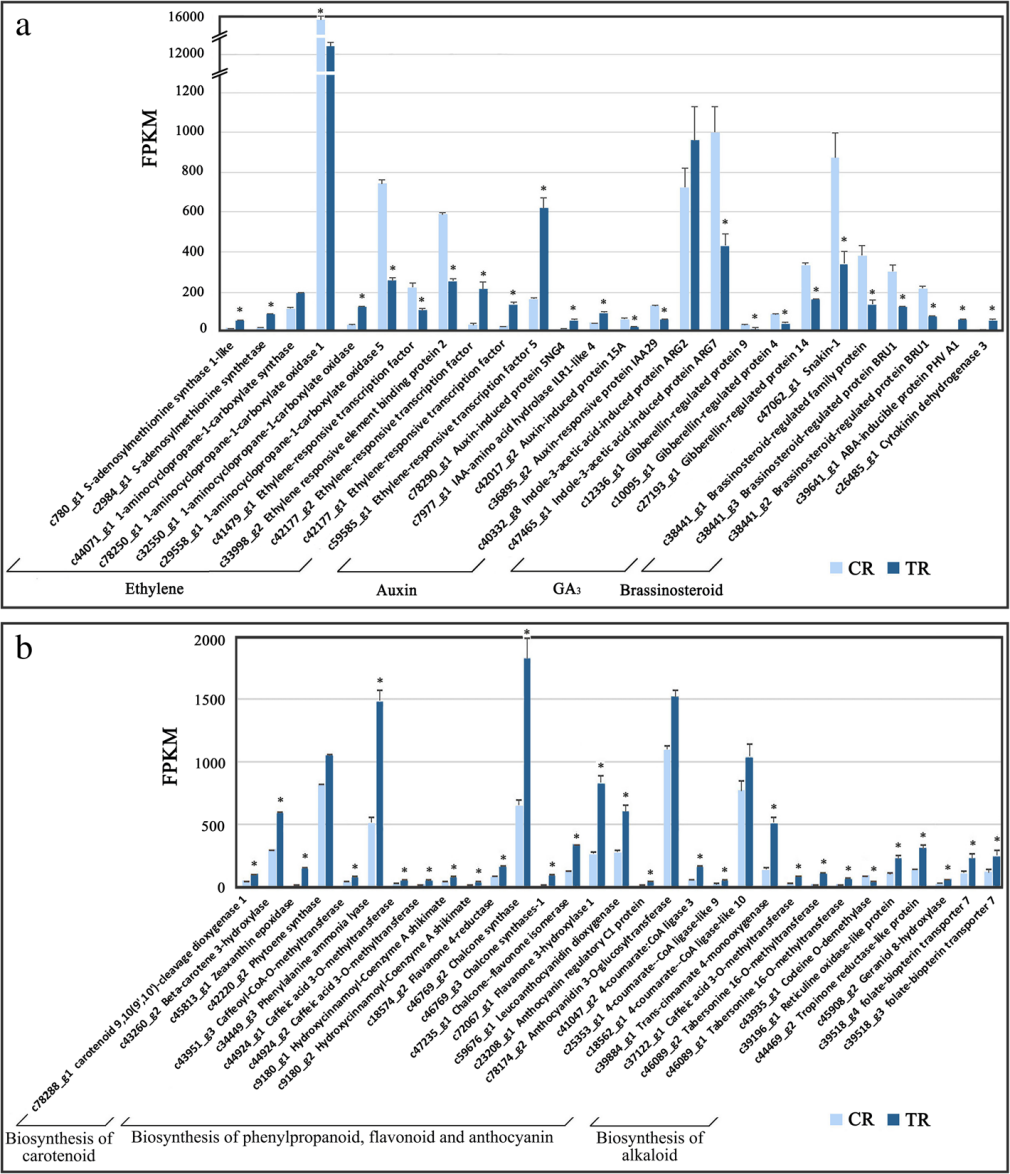
Differentially expressed genes (DEGs) related to plant hormone and secondary metabolite biosynthesis pathways of tree-ripe (TR) and commercial-ripe (CR) fig receptacles. **a** DEGs related to ethylene synthesis and other plant hormones. **b** DEGs related to secondary metabolite biosynthesis. *Significantly different at *p*-value (*p*-FDR) ≤ 0.05 and | log_2_FC | > = 1

DEGs related to ethylene synthesis, including two upregulated genes encoding s-adenosylmethionine synthetases (*c780_g1, c2984_g1*), two divergently expressed *ACO*s, and another *ACO* which had the highest FPKM value of all annotated genes and no significant difference in expression between the two ripening stages (Fig. 7a). Five DEGs encoding ERFs were found with divergent expression. The downregulated *CRF2* (*c33998_g2*)*-*encoded protein sequence showed 60.3% similarity to *Arabidopsis* cytokinin response factor. *ERF073* (*c41479_g1*) was downregulated, while *ERF5* (*c59585_g1*) and 2 *ERF110-like* were upregulated in TR fruit (Fig. 7a). These DEGs encode proteins that showed low similarity to ERFs of known function.

Significant expression changes were revealed in other plant hormone-signaling pathways, mainly the downstream components. Upregulation was revealed for four auxin pathway DEGs: two *auxin-induced protein 5NG4* genes, *IAA-amino acid hydrolase ILR1-like 4* and *indole-3-acetic acid-induced protein ARG2*; expression increments were also found with *cytokinin dehydrogenase 3* and *abscisic acid-inducible protein PHV A1*. Downregulation was found for *auxin-induced protein 15A, auxin-responsive protein IAA29* and *indole-3-acetic acid-induced protein ARG7*. Four genes related to gibberellin—three *gibberellin-regulated proteins* and *snakin*—were significantly repressed in TR vs. CR fruit. Similarly, three *brassinosteroid-regulated protein BRU1* genes were significantly downregulated (Fig. 7a).

In the secondary metabolism pathways, anthocyanidin 3-O-glucosyltransferase and chalcone synthase (CHS)—key enzymes affecting synthesis and accumulation of flavonoids, anthocyanins and other important secondary metabolites—was upregulated 1.42- and 1.39-fold in TR vs. CR fruit. 2-C-methyl-D-erythritol 2,4-cyclodiphosphate synthase, associated with biosynthesis isoprenoid, was upregulated 1.30-fold (Table 3). Isoprene is the main volatile component of the fruit and plays a number of important roles in development, plant defense and adaptation to environmental conditions [32].

DEGs in secondary metabolism pathways were mapped for biosynthesis of carotenoid, phenylpropanoid, flavonoid and anthocyanin, and biosynthesis of alkanoid. DEGs involved in carotenoid biosynthesis were all upregulated, including *carotenoid 9,10(9′,10′)-cleavage dioxygenase 1, beta-carotene 3-hydroxylase,* and *zeaxanthin epoxidase* (Fig. 7b). Most of the DEGs annotated in the phenylpropanoid-, flavonoid- and anthocyanin-biosynthesis pathways were upregulated in TR vs. CR fruit (Fig. 7b). This is in line with the increased flavonoid content during fig ripening [9]. Specifically, *CHS* was significantly upregulated with high FPKM, consistent with the proteomics result (Table 3).

In the category of alkanoid biosynthesis, *codeine O-demethylase* was the only downregulated gene of all of the DEGs associated with secondary metabolism. Expression of *tabersonine 16-O-methyltransferase, tropinone reductase-like protein* and *reticuline oxidase-like protein*, putatively involved in biosynthesis of indole alkaloids, tropine alkaloids, and benzo phenanthridine alkaloids, respectively, was upregulated in TR fruit (Fig. 7b). In addition, there was one *geraniol 8-hydroxylase*, putatively involved in monoterpenoid biosynthesis, and two *folate-biopterin transporter 7* which showed significant upregulation in the TR tissue. (Fig. 7b).

### Oxidoreductase and stress responses

Twelve DAPs were annotated as related to oxidoreductase activity. Nine of them were downregulated from CR to TR, the leading ones being peroxidase, glutathione S-transferase and D-3-phosphoglycerate dehydrogenase, while upregulation was mainly observed with L-ascorbate oxidase, monodehydroascorbate reductase and glutathione peroxidase (Table 3), illustrating active oxidoreduction homeostasis in the fig receptacle as ripening progresses.

**Fig. 8:**
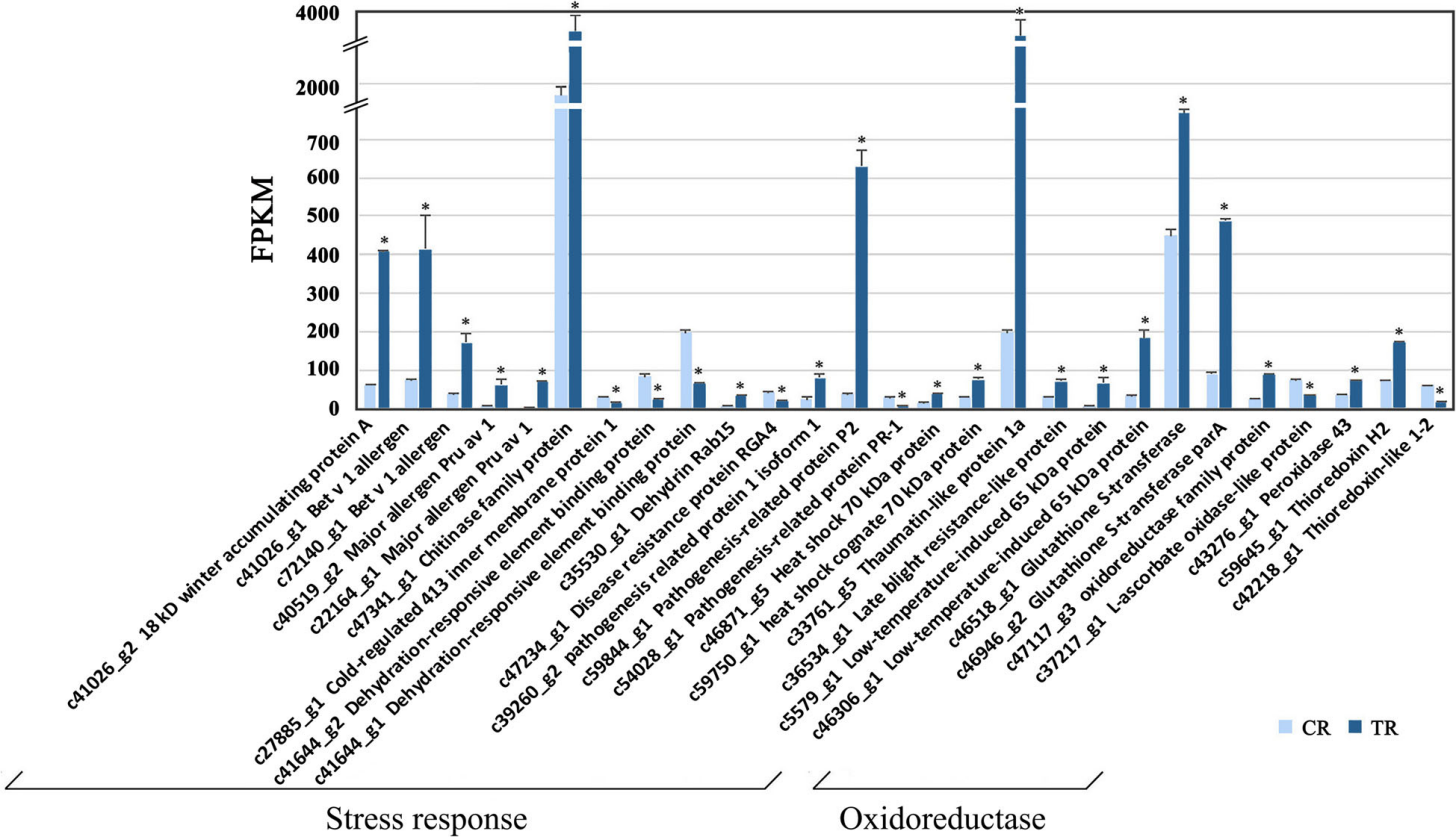
Differentially expressed genes related to stress responses and oxidoreductase of tree-ripe (TR) and commercial-ripe (CR) fig receptacles. *Significantly different at *p*-value (*p*-FDR) ≤ 0.05 and | log_2_FC | > = 1

DEGs related to the oxidoreductase were also mainly upregulated, including *glutathione s-transferase, oxidoreductase family protein* and *peroxidase*, which was in consistent with the proteomic result (Fig. 8, Table 3). *Thioredoxin (c42218_g1, c59645_g1)* showed divergent expression patterns. *L-ascorbate oxidase-like protein* showed downregulated, inconsistent with proteomic result (Fig. 8, Table 3)*.*

Six DAPs related to the stress response were identified, five upregulated, including two major allergens with high abundance, acidic endochitinase, dehydrin COR47 and pleiotropic drug resistance protein 1. Only one minor allergen was decreased in TR figs to 0.7 of their levels in CR figs (Table 3).

DEGs related to the stress response were mainly upregulated, especially those with high FPKM values, which was inconsistent with the proteome results. Of these, the gene encoding chitinase family protein, which had the highest FPKM value, showed 2.04-fold upregulated expression in TR fruit. DEGs annotated as encoding allergen homologs were all upregulated, including two *bet v 1 allergen homologs* (*c41026_g1, c72140_g1*) and two *major allergen Pru av 1 homologs* (*c22164_g1, c40519_g2*)*.* Blasting protein sequences revealed that *c41026_g1* and *c72140_g1* have 72.7 and 62.9% similarity, respectively, to the major pollen allergen Bet v 1-M/N of *Betula pendula*; *c22164_g1* and *c40519_g2* had 68.8 and 70.2% similarity, respectively, to the major allergen Pru av. 1 of *Prunus avium* (Additional file 10: Figure S7). The expression levels of the *major allergen Pru av 1 homolog* (*c22164_g1*)*, pathogenesis-related protein P2* and *thaumatin-like protein* changed notably, increasing more than 14 times in TR vs. CR. DEGs annotated as encoding pathogenesis-related proteins (*c54028_g1, c39260_g2, c59844_g1*) showed divergent expression patterns. Four genes related to stress response showed downregulated expression in TR fruit, including *cold-regulated 413 inner membrane protein 1,* two *dehydration-responsive element binding protein* genes (*c41644_g2, c41644_g1*) and *disease resistance protein RGA4* (Fig. 8).

## Discussion

The physiological state of a plant and its organs/tissues at different developmental stages is the result of fine regulation of complex systems. Significant physiological changes were noted in figs from the CR to TR stages, such as an increase in sugar content, changes in fruit firmness and color, and disappearance of latex flow. Combined analysis of the proteome and transcriptome provides a comprehensive understanding of the changes related to fruit quality and physiology between the two ripening stages. For instance, CHS and allergen homolog proteins were upregulated at both the protein and mRNA levels; the key enzyme in ethylene synthesis, ACO, was significantly downregulated at both the protein and mRNA levels.

Diverging protein and mRNA expression trends were also detected, such as for aquaporin and a large number of players in secondary metabolism. These inconsistencies might have been due to differences in mRNA and protein modification or turnover. Discrepancies in the throughput of the two omic methods also need to be considered, e.g., ‘order of magnitude’ whereby a lower number of proteins can be detected in the proteome analysis compared to their genes in the RNA-Seq analysis, isozymes can be missed, and difficulties in matching proteins with their corresponding RNAs.

### Latex-related proteins

Figs targeted for fresh consumption are often harvested at the CR stage. Due to the presence of latex, it is suggested that the laborers picking the fruit wear gloves. Latex may lead to skin irritation and corrosion, and consumers eating unripe figs with latex may be subject to oral cavity mucous membrane injury and allergic reactions [33, 34]. Latex contains ficins which belong to the papain subfamily in the family of cysteine proteases [30, 33]. The cysteine protease in latex participates in plant defense, and both papain and ficin are toxic to lepidopteran insects, causing growth stagnation or death of the larvae. This ʽtoxicity’ disappeared when papaya or fig leaves were sprayed with the cysteine protease inhibitor E64 [35]. Five different ficins (A–E) have been isolated from fig latex samples [30, 34]. In our study, ficins A, B, C and D were putatively identified from fig receptacle tissues, with ficin B and C being the most abundant isoforms in the leading fresh fig cv. Brown Turkey.

Latex gradually disappears in fig fruit from the CR to TR stage. Ficin, one of the most abundant proteins in figs, was also downregulated from CR to TR, consistent with physiological observations and previous reports [36]. The decrease in ficin levels, together with other cysteine proteinases in latex, from CR to TR might explain the fig’s total protein turnover and its edible quality at maturity. The transcript of cysteine proteinase RD21a, which may represent a ficin-encoding RNA, was detected in the transcriptome (Fig. 4).

### Sugar accumulation in fig

Sugar is of great importance to fig, as the major contributor to fresh fig taste, and as the main dry material in dried figs. Fig fruit accumulates sugar rapidly, in 3–5 days in favorable climates [37]. In our study, a very significant and rapid increase in sugar content was found from CR to TR.

Our proteomic data revealed upregulated sucrose synthases and SPS, two major players in sugar partitioning and accumulation. Sucrose synthase is usually present as more than one isoform in plants. The enzyme reversibly catalyzes sucrose to fructose and UDP-glucose, predominantly in sink tissues [38]. SPS resynthesizes sucrose from UDP-glucose and fructose 6-phosphate, and it plays an important role in sucrose accumulation and transport [39]. Elevated expression of specific isoforms of SPS has been reported in the late stage of apple development in line with sugar accumulation [40], whereas overexpression of SPS in tomatoes results in increased sucrose loading and transport rate [41]. Some of the important players in plant sugar translocation, partitioning and accumulation were not identified in our proteomic study, such as invertases and transporters. Their absence was probably due to their expression level, cellular localization, limitations of the extraction method or detection limits.

While at the transcriptional level, two highly transcribed sucrose synthase genes, and four *SPS*s were annotated by RNA-Seq, two invertase genes were found with very high expression (FPKM > 600) at both ripening stages: *bidirectional sugar transporter N3* with a FPKM value of more than 1000 and *raffinose synthase* with a value of about 500 (Additional file 9: Figure S6). Together with upregulated *amylases*, *hexokinase*s, *sugar transport protein 13* and *bidirectional sugar transporter SWEET12* (Fig. 5), our combined proteomic and transcriptomic results provide a network of key candidate genes for further study in fig sugar accumulation and its regulation.

### Fig fruit texture from CR to TR

Fruit texture and fruit skin strength are important attributes in fruit production and transport, as they largely determine the methods used for fruit harvest, postharvest sorting, packaging, and transportation. Fruit texture and skin strength are also important in consumers’ assessment of fruit quality and preferences [4, 42]. Fig texture and skin strength are remarkably more resistant in CR vs. TR fruit to harvesting, transport and shelf handling.

Decreased fig fruit hardness was mainly due to changes in receptacle texture and physiological status. It has long been known that structural changes in pectin, hemicellulose and cellulose are responsible for the alterations in cell-wall structure during fruit ripening-related loss of firmness [43]. In cell-wall modifications, pectolytic enzymes cleave or modify the nature of the polysaccharide backbone or remove neutral sugars from branched side chains, while non-pectolytic enzymes are responsible for hemicellulose modifications [44]. Our study showed upregulation from the CR to TR stage of non-pectolytic enzymes, such as expansins, endoglucanase and xyloglucan endotransglycosylase, and of pectolytic enzymes, mainly beta-galactosidase and polygalacturonases. The results revealed key candidate players in late-stage fig softening, which is strongly connected to fig transportability and shelf life. Further study of the regulation of these candidate enzymes’ expression could ultimately lead to highly storable and transportable cultivars, as has been achieved in tomato breeding in the last 30 years [45].

### Expression of genes involved in the pathways of ethylene and other plant hormones

Plant hormones are involved in every aspect of plant growth and development. In fruit ripening, ethylene and abscisic acid are well known to trigger ripening and coordinate with other plant hormones in the whole fruit-ripening process [46, 47]. Fig has been regarded as a climacteric fruit, mainly female flower developed tissue, while the receptacle carried the non-climacteric features [20]. Our study demonstrated active expression regulation of ethylene-pathway proteins and genes during the ripening process of fig receptacle tissues (Table 3, Fig. 7) from CR to TR. ACO was significantly downregulated at both the protein and mRNA levels; SAS was also downregulated at the protein level; its upregulation at the RNA level can be explained by the widespread post-transcriptional regulation of ethylene biosynthesis [48, 49]. Our RNA-Seq results identified a large number of *ERF* genes. *ERF5*, with a high FPKM value, significantly increased in TR fig, and may be involved in regulating the stress response [50, 51]. Downregulation of *ERF073* may serve to activate nuclear transcription in the hypoxia response [52]. From CR to TR, fig receptacle diversely expressed ethylene-response factors and decreased ethylene synthesis ability, potentially endorse the leading role of ethylene in the ripening process of fig receptacle.

Other plant hormone pathway components also revealed active expression changes from CR to TR. The genes encoding gibberellin-related proteins and brassinosteriod-regulated proteins were all downregulated (Fig. 7a). However, their functions are not yet clear. Some studies have demonstrated that *GASA14* regulates leaf expansion and abiotic stress resistance by modulating accumulation of reactive oxygen species [53]. Mutated *GASA4* loses its redox activity and the ability to promote gibberellin responses [54]. *SN1* silencing affects cell division, leaf primary metabolism, and cell-wall composition in potato plants [55].

## Conclusions

There is a remarkable increase in fig size, weight, sugar content and flavor from CR to TR stages, accompanied by a decrease in fruit firmness. Combined quantitative proteomic and transcriptomic results demonstrate differentially regulated proteins and genes related to ficins, sugar accumulation, cell-wall modification, ethylene pathway and other biological processes involved in fruit quality and ripening. Our integrative multi-omic results provide valuable information for understanding the process of fig ripening, specifically, the underlined biomolecular base of the final formation of optimal consumption quality, which could illuminate fig cultivar breeding and innovation to best fit the consumer and market requirements.

## Additional files

Additional file 1: **Table S1.** Primer sequences for genes used for verification of digital gene-expression results by quantitative real-time PCR. (DOCX 16 kb)

Additional file 2: **Table S2.** Proteinase- and protein turnover-related differentially expressed genes (DEGs) in tree-ripe (TR) and commercial-ripe (CR) Brown Turkey figs. (DOCX 29 kb)

Additional file 3: **Table S3.** All identified proteins annotated in Moraceae and local RNA-Seq assembly dataset databases. (XLSX 146 kb)

Additional file 4: **Figure S1.** Workflow of the experimental procedure. (DOCX 6090 kb)

Additional file 5: **Figure S2.** Workflow outlining protein matches in databases. (DOCX 3344 kb)

Additional file 6: **Figure S3.** Validation of 15 genes with high FPKM by qRT-PCR analysis. CR, commercial ripe; TR, tree ripe. (DOCX 4277 kb)

Additional file 7: **Figure S4.** Protein sequence alignment of ficin A, B, C, D (A0A182DW06, 08, 09 and 11_FICCA, respectively), cysteine proteinase RD21a, RD19a (RD21a and RD19a_ARATH, respectively), and thiol protease aleurain (ALEU_ARATH). Asterisk, identical positions; one dot, weakly similar positions; two dots, similar positions. (DOCX 5246 kb)

Additional file 8: **Figure S5.** Protein sequence alignment of sucrose synthase (SS) and sucrose-phosphate synthase (SPS). **a** *Ficus carica* SS sequence alignment with that of *Citrus nobilis* var. Unshiu (Q9SLY2_CITUN) and *Vitis vinifera* (A5C6H7_VITVI). **b** *Ficus carica* SPS sequence alignment with that of *Citrus nobilis* var. Unshiu (SPSA1_CITUN) and *Prunus persica* (I1W1U0_PRUPE). Asterisk, identical positions; one dot, weakly similar positions; two dots, similar positions. (DOCX 4414 kb)

Additional file 9: **Figure S6.** Essential but non-significant DEGs related to sugar accumulation. CR, commercial ripe; TR, tree ripe. (DOCX 3003 kb)

Additional file 10: **Figure S7.** Protein sequence alignment of allergenic proteins and the identified homologs from fig. PRU1_PRUAV: major allergen Pru av. 1 of *Prunus avium*; BEV1M_BETPN: major pollen allergen Bet v 1-M/N of *Betula pendula*; ALTA7_ALTAL: minor allergen Alt a 7 of *Alternaria alternata*. Two dots, similar positions. (DOCX 4251 kb)

## Abbreviations

ACO: 1-aminocyclopropane-1-carboxylate oxidase
CHS: Chalcone synthase
CR: Commercial-ripe
DAPs: Differential abundance proteins
DEGs: Differentially expressed genes
ERFs: Ethylene-response factors
FA: Formic acid
FC: Fold-change
FDR: False discovery rate
FPKM: Fragments per kilobase of exon model per million mapped reads
GO: Gene ontology
HCD: Higher-energy collisional dissociation
iTRAQ: Isobaric tags for relative and absolute quantification
KEGG: Kyoto Encyclopedia of Genes and Genomes
LC: Liquid chromatography
MS: Mass spectrometer
NCBI: National Coalition Building Institute
NCE: Normalized collision energy
PCR: Polymerase Chain Reaction
RNA-Seq: RNA sequencing
RSEM: RNA-Seq by Expectation-Maximization
SDS-PAGE: Sodium dodecyl sulfate polyacrylamide gel electrophoresis
SNR: Signal-to-noise ratio
SPS: Sucrose-phosphate synthase
TCA: Trichloroacetic acid
TEAB: Triethylamine borane
TR: Tree-ripe
