# Supplementary material for: Proteome and transcriptome analyses reveal key molecular differences between quality parameters of commercial-ripe and tree-ripe fig (Ficus carica L.)
Source: BMC Plant Biol. 2019 Apr 16;19:146. doi: 10.1186/s12870-019-1742-x (PMC6469076; doi:10.1186/s12870-019-1742-x)
Supplement: Supplementary file 1 — Table S1. Primer sequences for genes used for verification of digital gene-expression results by quantitative real-time PCR. (DOCX 16 kb) [file 12870_2019_1742_MOESM1_ESM.docx]

| Gene ID | Gene name | Sequence (5ˊ→3ˊ)-F | Sequence(5ˊ→3ˊ)-R |
| --- | --- | --- | --- |
| c42134_g1 | *Cysteine proteinase RD19a* | GTACGACGGGAGCACTGGAA | CCTCCTCACGCTGTAGTCCG |
| c42827_g1 | *Cysteine proteinase RD21a* | CTGGGGTGAGGACGGCTATG | AGGCCTCCATCGCAATTCCA |
| c26558_g1 | *Sucrose synthase 2* | CGTGACATGTGGCGAACGTC | ATCAGCGCAGACTAACCGGG |
| c47307_g1 | *Major latex allergen Hev b 5* | GCAACAACAACAGTGCCGGA | TGTGGTTGCTGCGGGTTCTA |
| c23588_g1 | *Bidirectional sugar transporter N3* | GCAGCACCCTTGAGCATTGT | GAAGTGGCTAGTGTGCGCAG |
| c25248_g1 | *Beta-amylase 1, chloroplastic* | GTGCTACGCCGACTTCATGC | CCTGCTCTGGGTACGAAGGG |
| c33895_g4 | *Probable pectate lyase 20* | TGAGCTCCGAACCATGGCAT | CCGAGCGACCATGATCCAGT |
| c29668_g1 | *Endoglucanase 24* | GATCCTCCTTGCCCTCGGTC | GTGGGCTCGGACTTCCTGAA |
| c39769_g1 | *Dehydrin COR47* | TTGAGACCAAGGATCGCGGG | GGACCGGTGAAGCTTCTCGA |
| c33761_g5 | *Thaumatin-like protein 1a* | CTCTTGCACCCAATGACGCC | GGTTCAACTTGCCGGTGTCG |
| c32550_g1 | *1-Aminocyclopropane-1-carboxylate oxidase* | GACGATGGAGTGGCGCATTG | TACCCTCCATGCCCCAAACC |
| c632_g1 | *Auxin-induced protein 5NG4* | CCCATCAGAAGCCTCTCCCG | TGGGAGTGTGATTGGAGCGG |
| c59585_g1 | *Ethylene-responsive transcription factor 5* | CGTCACGCAGCTACTCTCCA | GGAAGTTACCCTCTCCGGCG |
| c27193_g1 | *Gibberellin-regulated protein 14* | CACACGCGTCCACTTTTGCT | AGAGGAGCAACACGGCCATT |
| c38162_g1 | *Pectinesterase 3* | TCGACGGCACTCCCACTTTT | GCGGTAGAATTGGCGGTTGG |
| c43062_g4 | *Actin-7* | CCCATGCCATCCTCCGTCTT | CCTGCTCGTAGTCCAAGGCA |

**Additional file 1: Table S1.** Primer sequences of genes used for verification of digital gene-expression results by quantitative real-time PCR.
