# Supplementary material for: Proteome and transcriptome analyses reveal key molecular differences between quality parameters of commercial-ripe and tree-ripe fig (Ficus carica L.)
Source: BMC Plant Biol. 2019 Apr 16;19:146. doi: 10.1186/s12870-019-1742-x (PMC6469076; doi:10.1186/s12870-019-1742-x)
Supplement: Supplementary file 2 — Table S2. Proteinase- and protein turnover-related differentially expressed genes (DEGs) in tree-ripe (TR) and commercial-ripe (CR) Brown Turkey figs. (DOCX 29 kb) [file 12870_2019_1742_MOESM2_ESM.docx]

**Additional file 2: Table S2.** Proteinase- and protein turnover-related differentially expressed genes (DEGs) in commercial-ripe (CR) and tree-ripe (TR) figs.

| Seq_id | CR_mean  FPKM | | TR_mean  FPKM | Fold change,  Log_2_(FC) | *p*-value | NR_description |
| --- | --- | --- | --- | --- | --- | --- |
| Proteinase | |  | | |  |  |
| c42104_g1 | 8.25 | | 1.61 | -2.29 | 2.67E-24 | Subtilisin-like protease |
| c56808_g1 | 1.43 | | 0.29 | -1.98 | 5.03E-03 | Microsomal signal peptidase |
| c27443_g2 | 1.35 | | 0.32 | -1.81 | 2.98E-04 | Aspartic proteinase nepenthesin-2 |
| c20890_g1 | 14.48 | | 4.34 | -1.72 | 3.70E-06 | Serine carboxypeptidase-like 7 |
| c32240_g2 | 35.1 | | 11.47 | -1.6 | 1.72E-13 | Subtilisin-like protease |
| c47131_g1 | 54.88 | | 18.41 | -1.57 | 6.07E-31 | Germination-specific cysteine protease 1 |
| c23625_g1 | 48.3 | | 16.36 | -1.56 | 1.26E-32 | Aspartic proteinase nepenthesin-1 |
| c32240_g1 | 50.95 | | 17.34 | -1.55 | 7.89E-23 | Subtilisin-like protease |
| c30484_g2 | 38.56 | | 15.22 | -1.34 | 3.59E-09 | Aspartic proteinase nepenthesin-2 |
| c14360_g1 | 7.36 | | 3.05 | -1.24 | 4.70E-04 | Xylem cysteine proteinase 1 |
| c40157_g1 | 23.94 | | 10.68 | -1.16 | 5.21E-17 | Proline iminopeptidase |
| c42104_g2 | 7.96 | | 3.51 | -1.16 | 6.66E-09 | Subtilisin-like protease |
| c45672_g1 | 42.84 | | 19.56 | -1.13 | 1.29E-18 | Subtilisin-like protease |
| c30484_g1 | 27.42 | | 13.23 | -1.05 | 9.74E-05 | Aspartic proteinase nepenthesin-2 |
| c10393_g1 | 4.86 | | 2.34 | -1.02 | 2.01E-03 | Subtilisin-like protease |
| c33979_g1 | 1.17 | | 3.42 | 1.47 | 3.74E-08 | Subtilisin-like protease |
| c23262_g1 | 4.14 | | 12.01 | 1.51 | 3.09E-12 | Metalloendoproteinase 1 |
| c35672_g1 | 0.16 | | 0.87 | 1.91 | 7.67E-03 | Protease Do-like 8, chloroplastic |
| c31748_g1 | 0.36 | | 1.84 | 2.06 | 4.51E-05 | Aspartic proteinase |
| c41473_g1 | 0 | | 6.69 | 6.09 | 9.91E-24 | Metalloendoproteinase 1 |
| Proteinase inhibitors | | |  |  |  |  |
| c44792_g1 | 19.44 | | 67.93 | 1.8 | 1.85E-27 | Alpha-amylase/subtilisin inhibitor |
| c21517_g1 | 0.95 | | 7.8 | 2.91 | 2.17E-11 | Endogenous alpha-amylase/subtilisin inhibitor |
| c29537_g1 | 38.39 | | 294.1 | 2.93 | 1.43E-91 | Alpha-amylase/subtilisin inhibitor |
| c5096_g1 | 4.28 | | 34.96 | 3 | 7.30E-12 | Proteinase inhibitor |
| c36160_g1 | 2.35 | | 29.35 | 3.59 | 3.32E-26 | Alpha-amylase/subtilisin inhibitor |
| c5096_g1 | 4.28 | | 34.96 | 3 | 7.30E-12 | Alpha-amylase/subtilisin inhibitor |
| c43066_g1 | 0.27 | | 4 | 3.47 | 4.38E-11 | Serine protease inhibitor 2 |
| c29537_g1 | 38.39 | | 294.1 | 2.93 | 1.43E-91 | Alpha-amylase/subtilisin inhibitor |
| Ribosomal proteins | | |  |  |  |  |
| c53793_g1 | 0 | | 1.95 | 4.36 | 5.90E-05 | Ribosomal protein S7, mitochondrion |
| c40657_g2 | 0.47 | | 1.43 | 1.44 | 7.32E-03 | 40S ribosomal protein S3 |
| c43714_g1 | 0.16 | | 3.59 | 3.85 | 8.20E-05 | 40S ribosomal protein S5 |
| c32039_g1 | 0 | | 2.05 | 4.43 | 1.17E-06 | 40S ribosomal protein S5a |
| c78975_g1 | 0 | | 2.1 | 4.46 | 1.39E-07 | 40S ribosomal protein S6 |
| c24683_g1 | 0 | | 2.13 | 4.48 | 3.90E-04 | 40S ribosomal protein S13 |
| c35025_g1 | 0 | | 2.24 | 4.55 | 1.81E-07 | 40S ribosomal protein S4 |
| c27551_g1 | 0 | | 2.32 | 4.6 | 7.27E-08 | 40S ribosomal protein SA |
| c37304_g1 | 0 | | 2.59 | 4.75 | 1.28E-03 | 40S ribosomal protein S14 |
| c33353_g1 | 0 | | 3.34 | 5.1 | 2.39E-07 | 40S ribosomal protein S19a |
| c35604_g1 | 0 | | 3.68 | 5.24 | 2.11E-08 | 40S ribosomal protein S7 |
| c29184_g1 | 0 | | 3.82 | 5.29 | 3.08E-09 | 40S ribosomal protein S8 |
| c27256_g1 | 0 | | 3.91 | 5.32 | 1.74E-06 | 40S ribosomal protein S23 |
| c31865_g1 | 4.17 | | 1.87 | -1.12 | 9.85E-03 | 50S ribosomal protein L21, chloroplast |
| c43533_g2 | 4.55 | | 1.76 | -1.32 | 6.05E-04 | 50S ribosomal protein L4, chloroplast |
| c40100_g2 | 0 | | 1.04 | 3.52 | 9.52E-06 | 60S acidic ribosomal protein P0 |
| c38337_g1 | 0 | | 1.49 | 3.99 | 7.80E-05 | 60S ribosomal protein L13 |
| c12901_g1 | 0 | | 1.64 | 4.12 | 1.46E-04 | 60S ribosomal protein L7a |
| c21720_g1 | 0 | | 1.7 | 4.17 | 2.52E-06 | 60S ribosomal protein L7 |
| c33849_g1 | 0 | | 1.87 | 4.3 | 1.19E-04 | 60S ribosomal protein L24 |
| c53793_g1 | 0 | | 1.95 | 4.36 | 5.90E-05 | 60S ribosomal protein L9 |
| c37774_g1 | 0 | | 1.98 | 4.38 | 2.08E-04 | 60S ribosomal protein L11 |
| c40400_g2 | 0 | | 2.27 | 4.57 | 5.78E-05 | 60S ribosomal protein L27a |
| c40083_g1 | 0 | | 2.39 | 4.64 | 3.17E-05 | 60S ribosomal protein L12 |
| c32475_g1 | 0 | | 2.43 | 4.66 | 5.23E-05 | 60S ribosomal protein L28 |
| c34556_g1 | 0 | | 2.6 | 4.75 | 3.95E-14 | 60S ribosomal protein L3 |
| c12862_g1 | 0 | | 2.68 | 4.8 | 4.44E-06 | 60S ribosomal protein L14 |
| c15318_g1 | 0 | | 2.82 | 4.87 | 2.59E-09 | 60S ribosomal protein L6 |
| c599_g1 | 0 | | 2.95 | 4.93 | 1.46E-05 | 60S ribosomal protein L34 |
| c11186_g1 | 0 | | 3.25 | 5.06 | 8.36E-06 | 60S ribosomal protein L13a |
| c38111_g1 | 0 | | 4.54 | 5.54 | 1.55E-08 | 60S acidic ribosomal protein P2 |
| Ubiquitin-related proteins | | | | | | |
| c13531_g1 | 8.65 | | 3.09 | -1.46 | 6.09E-12 | U-box domain-containing protein 15 |
| c26584_g1 | 13.83 | | 6.29 | -1.12 | 1.54E-07 | E3 ubiquitin-protein ligase MGRN1 |
| c45908_g1 | 2.58 | | 1.22 | -1.03 | 1.84E-03 | Ubiquitin carboxyl-terminal hydrolase 12 |
| c18577_g1 | 16.32 | | 33.03 | 1.01 | 1.31E-11 | Putative E3 ubiquitin-protein ligase BAH1-like 1 |
| c46253_g1 | 8.36 | | 17.72 | 1.08 | 6.53E-04 | E3 ubiquitin-protein ligase RING1-like protein |
| c30230_g1 | 0.17 | | 0.65 | 1.47 | 2.20E-04 | Putative E3 ubiquitin-protein ligase LIN-1 |
| c43896_g1 | 3.47 | | 10.45 | 1.57 | 4.48E-12 | E3 ubiquitin-protein ligase ATL6 |
| c32371_g1 | 31.52 | | 95.38 | 1.59 | 1.05E-35 | U-box domain-containing protein 20 |
| c49304_g1 | 0.79 | | 2.97 | 1.78 | 6.78E-03 | Putative E3 ubiquitin-protein ligase HERC1 |
| c26605_g1 | 1.43 | | 5.62 | 1.9 | 8.74E-08 | E3 ubiquitin-protein ligase ATL41 |
| c60224_g1 | 1.45 | | 6.41 | 2.08 | 8.97E-06 | E3 ubiquitin-protein ligase ATL41 |
| c18272_g1 | 0.15 | | 1.01 | 2.18 | 8.41E-05 | Ubiquitin-protein transferase |
| c41104_g1 | 0.56 | | 2.88 | 2.18 | 1.59E-03 | E3 ubiquitin-protein ligase RHA2A |
| c34895_g1 | 2.44 | | 11.47 | 2.19 | 7.07E-18 | Ubiquitin-protein transferase |
| c38586_g2 | 0.32 | | 2.26 | 2.51 | 1.21E-04 | E3 ubiquitin-protein ligase ATL6 |
| c41421_g3 | 0.14 | | 1.33 | 2.57 | 6.73E-04 | E3 ubiquitin-protein ligase RGLG2 |
| c73239_g1 | 0.12 | | 1.83 | 3.11 | 2.98E-09 | E3 ubiquitin-protein ligase |
| c31420_g1 | 0.14 | | 12.61 | 5.75 | 2.08E-14 | E3 ubiquitin-protein ligase RHA2A |
| Proteosome-related proteins | | | | | |  |
| c53874_g1 | 13.71 | | 2.65 | -2.33 | 1.33E-02 | Proteasome, ubiquitin receptor RAD23c |
| c45958_g4 | 2.67 | | 6.96 | 1.35 | 8.07E-11 | Proteasome core complex |
| c36464_g1 | 0.35 | | 1.17 | 1.5 | 8.31E-03 | 26S proteasome non-ATPase regulatory subunit 10 |
