# Supplementary figures and images for: Proteome and transcriptome analyses reveal key molecular differences between quality parameters of commercial-ripe and tree-ripe fig (Ficus carica L.)
Source: BMC Plant Biol. 2019 Apr 16;19:146. doi: 10.1186/s12870-019-1742-x (PMC6469076; doi:10.1186/s12870-019-1742-x)

Additional file 4: Figure S1


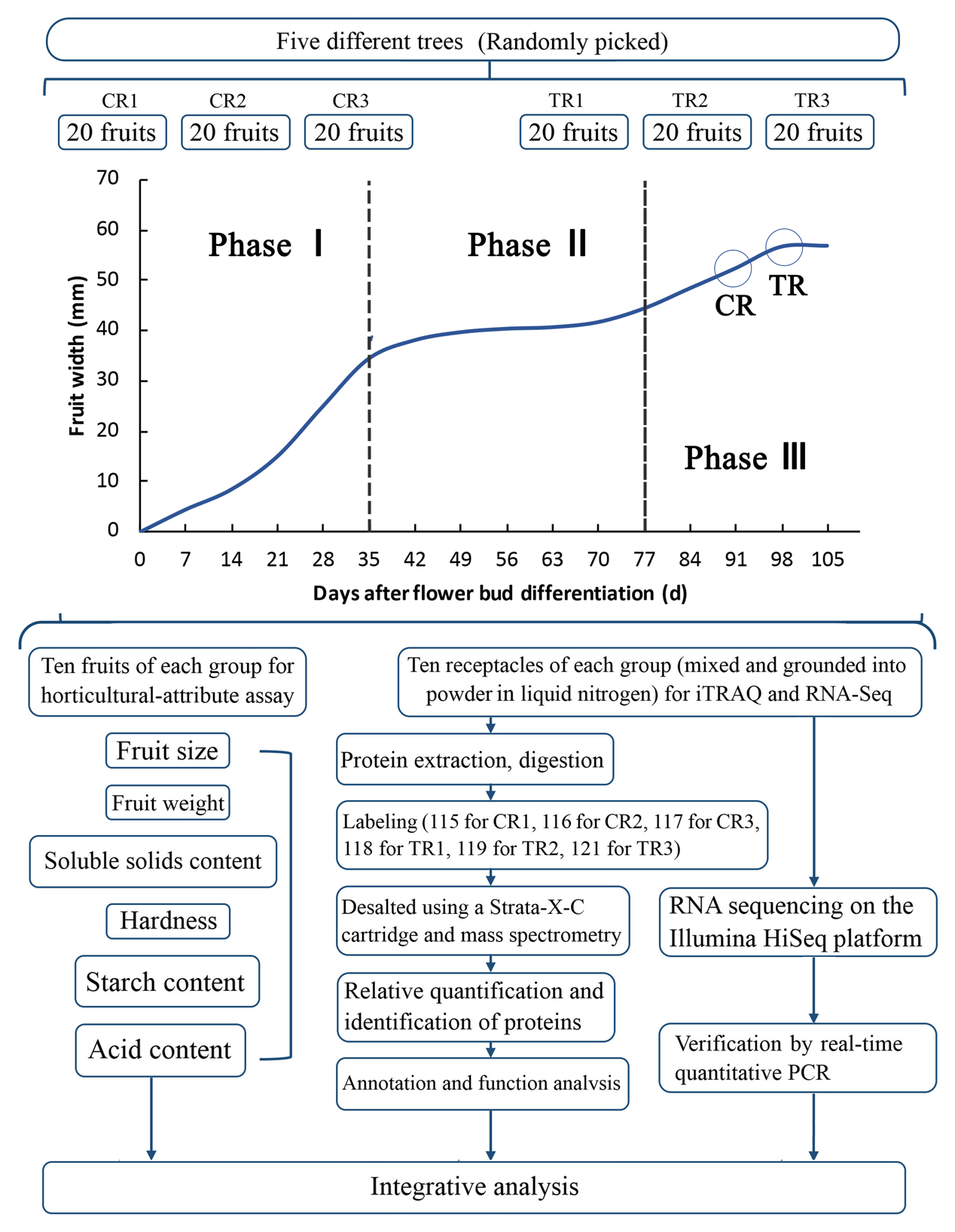

Supplement: Supplementary file 4 — Figure S1. Workflow of the experimental procedure. (DOCX 6090 kb) [file 12870_2019_1742_MOESM4_ESM.docx]

Additional file 5: Figure S2


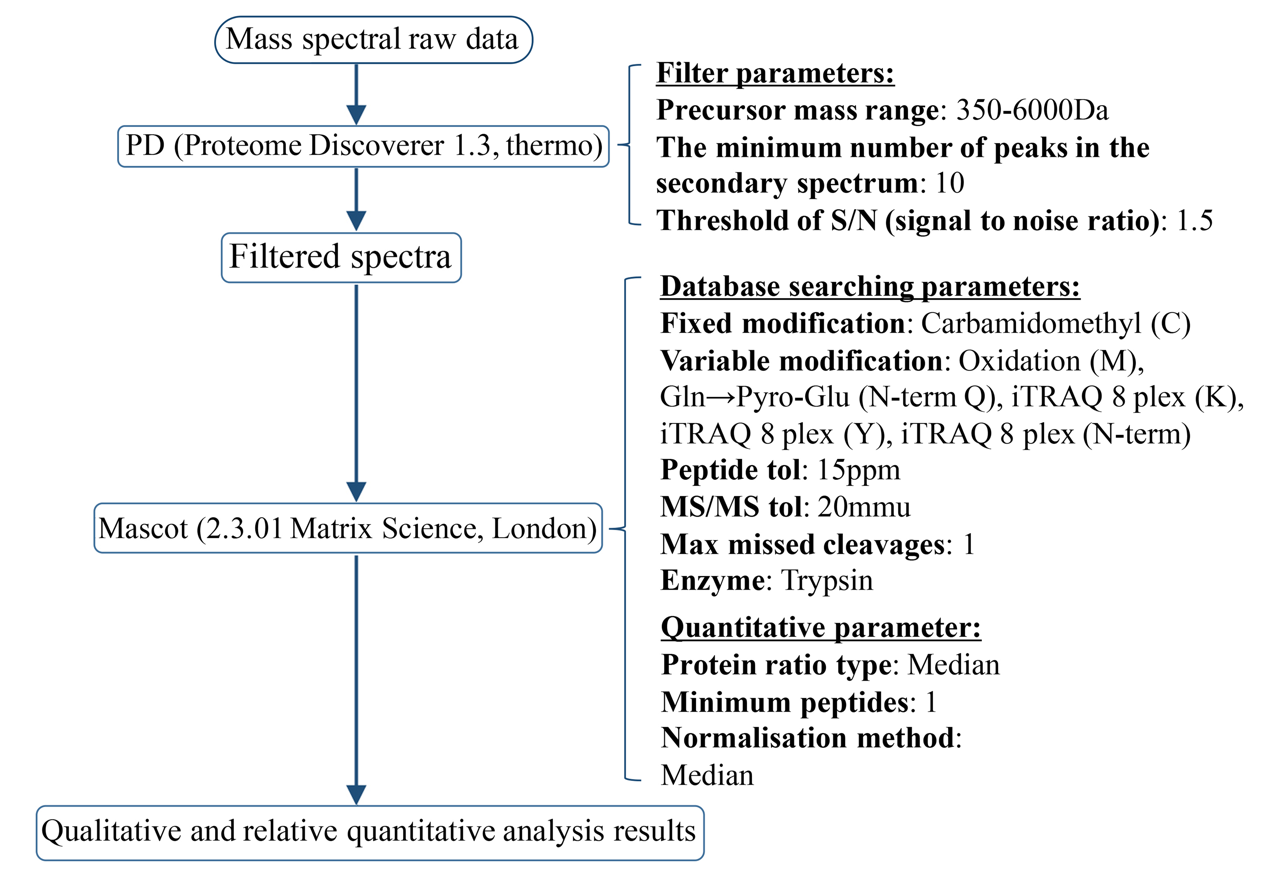

Supplement: Supplementary file 5 — Figure S2. Workflow outlining protein matches in databases. (DOCX 3344 kb) [file 12870_2019_1742_MOESM5_ESM.docx]

Additional file 6: Figure S3


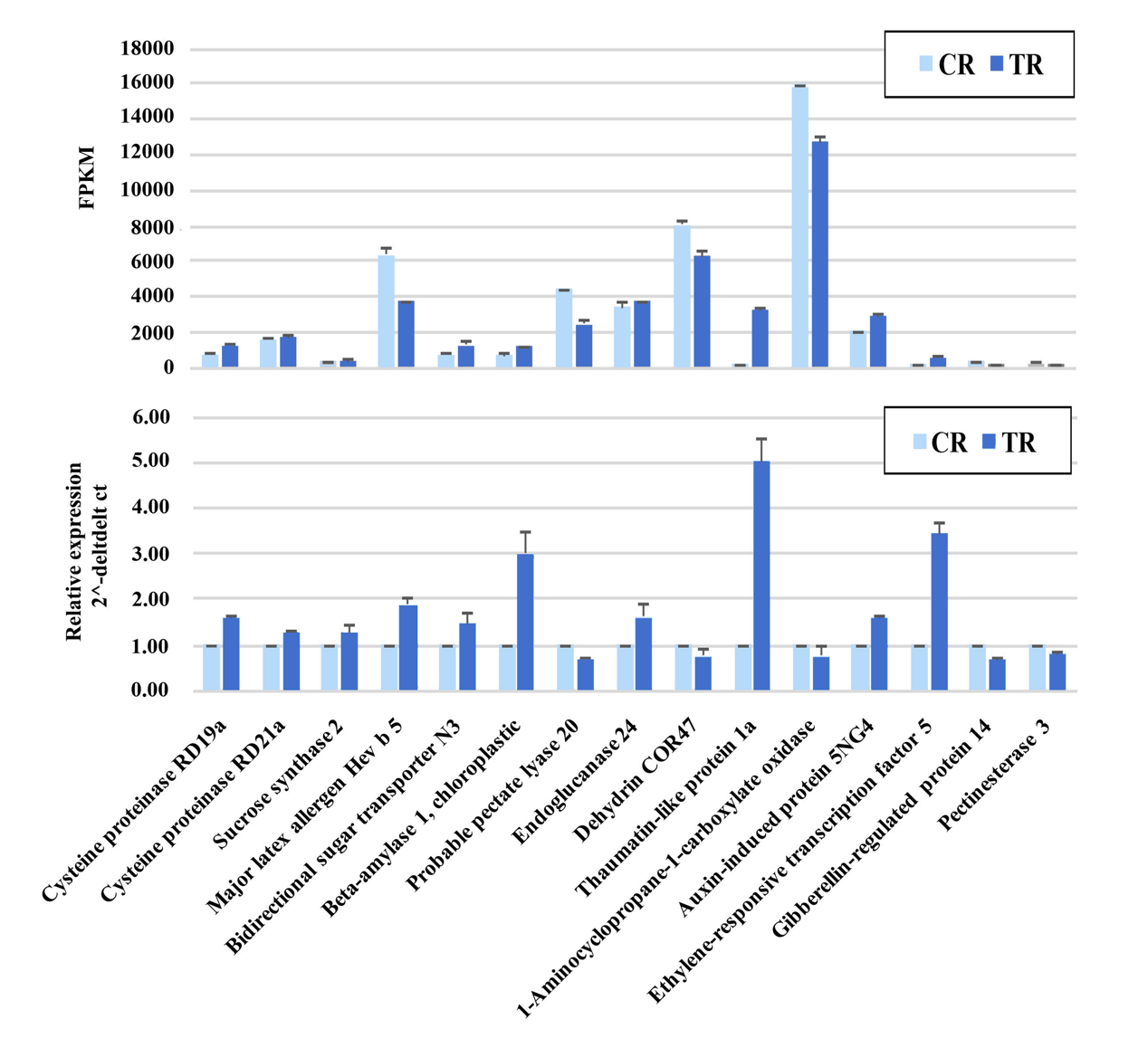

Supplement: Supplementary file 6 — Figure S3. Validation of 15 genes with high FPKM by qRT-PCR analysis. CR, commercial ripe; TR, tree ripe. (DOCX 4277 kb) [file 12870_2019_1742_MOESM6_ESM.docx]

Additional file 7: Figure S4


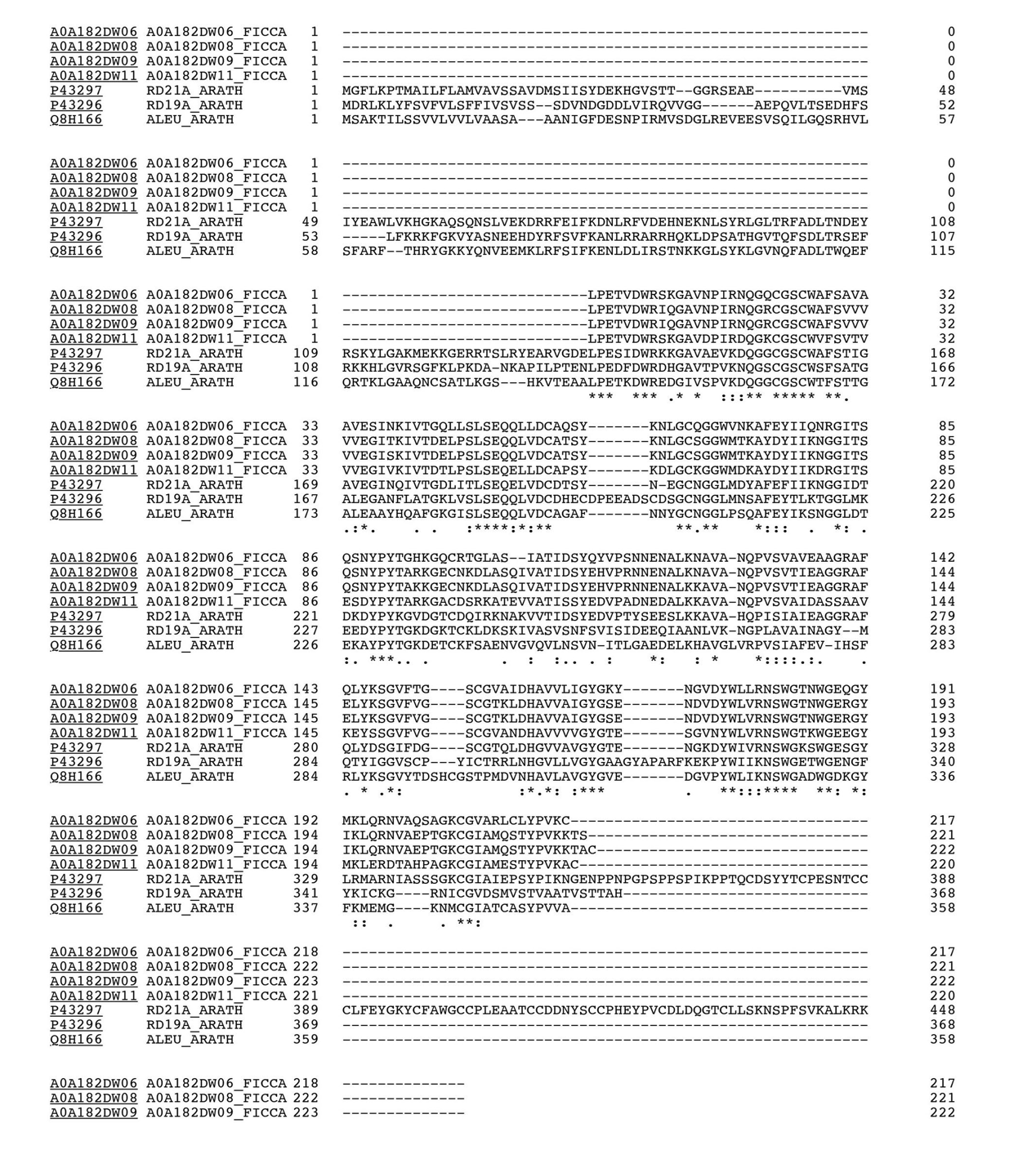

Supplement: Supplementary file 7 — Figure S4. Protein sequence alignment of ficin A, B, C, D (A0A182DW06, 08, 09 and 11_FICCA, respectively), cysteine proteinase RD21a, RD19a (RD21a and RD19a_ARATH, respectively), and thiol protease aleurain (ALEU_ARATH). Asterisk, identical positions; one dot, weakly similar positions; two dots, similar positions. (DOCX 5246 kb) [file 12870_2019_1742_MOESM7_ESM.docx]

Additional file 8: Figure S5


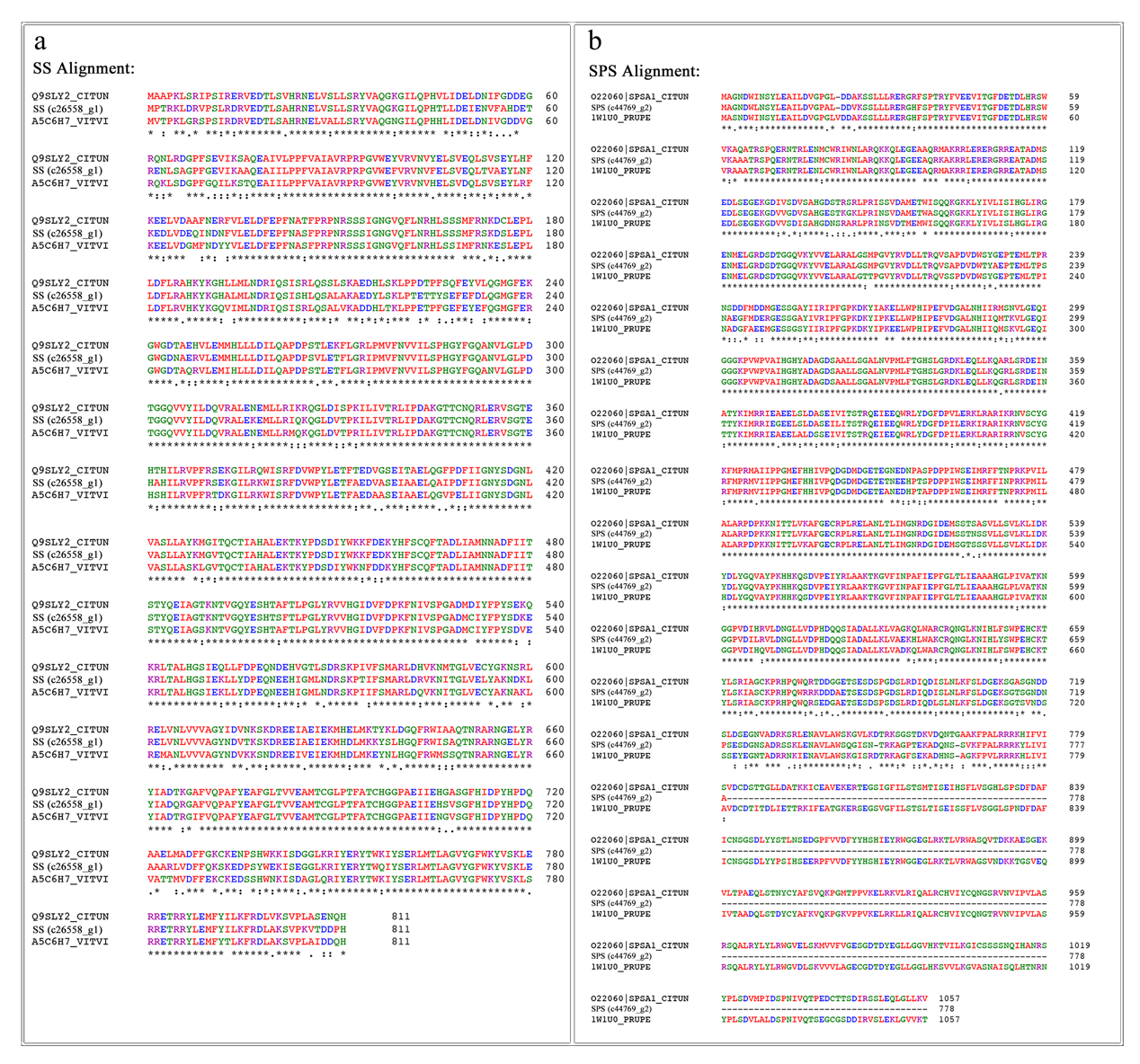

Supplement: Supplementary file 8 — Figure S5. Protein sequence alignment of sucrose synthase (SS) and sucrose-phosphate synthase (SPS). a Ficus carica SS sequence alignment with that of Citrus nobilis var. Unshiu (Q9SLY2_CITUN) and Vitis vinifera (A5C6H7_VITVI). b Ficus carica SPS sequence alignment with that of Citrus nobilis var. Unshiu (SPSA1_CITUN) and Prunus persica (I1W1U0_PRUPE). Asterisk, identical positions; one dot, weakly similar positions; two dots, similar positions. (DOCX 4414 kb) [file 12870_2019_1742_MOESM8_ESM.docx]

Additional file 9: Figure S6


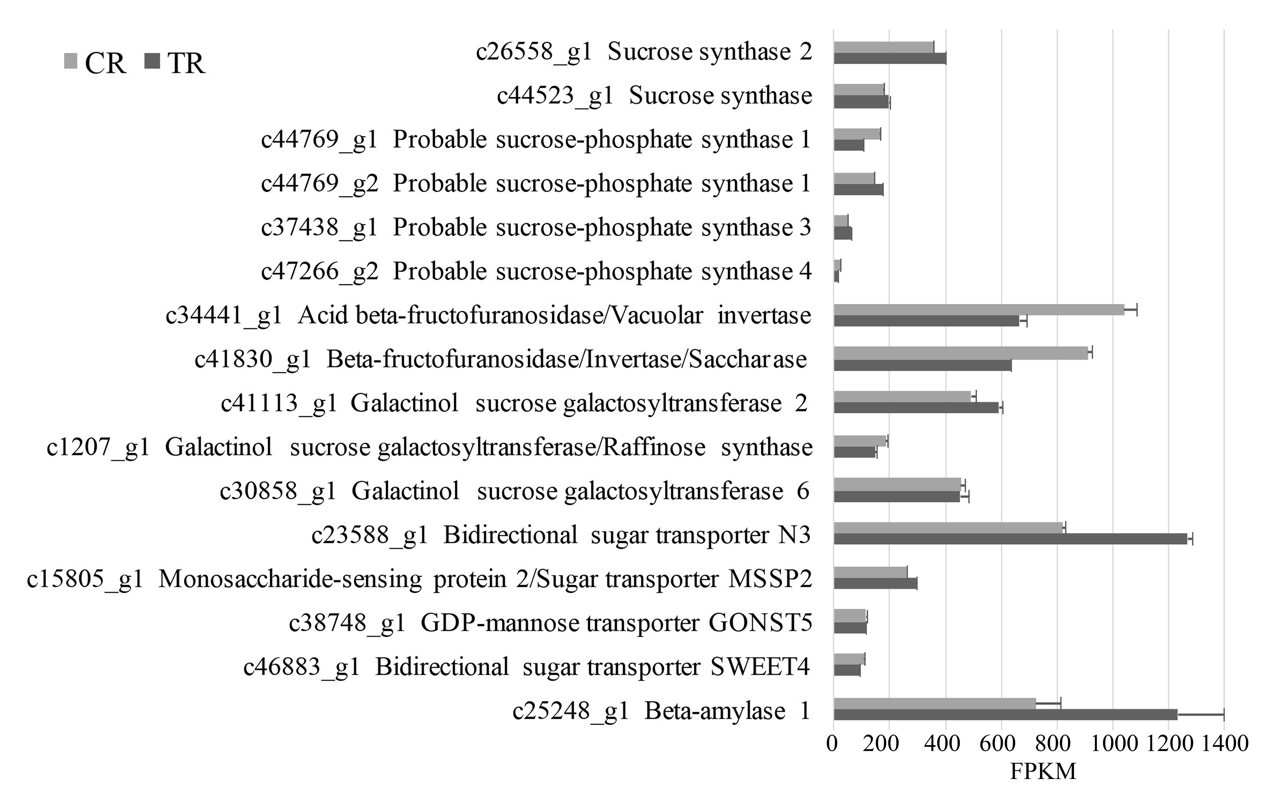

Supplement: Supplementary file 9 — Figure S6. Essential but non-significant DEGs related to sugar accumulation. CR, commercial ripe; TR, tree ripe. (DOCX 3003 kb) [file 12870_2019_1742_MOESM9_ESM.docx]

Additional file 10: Figure S7


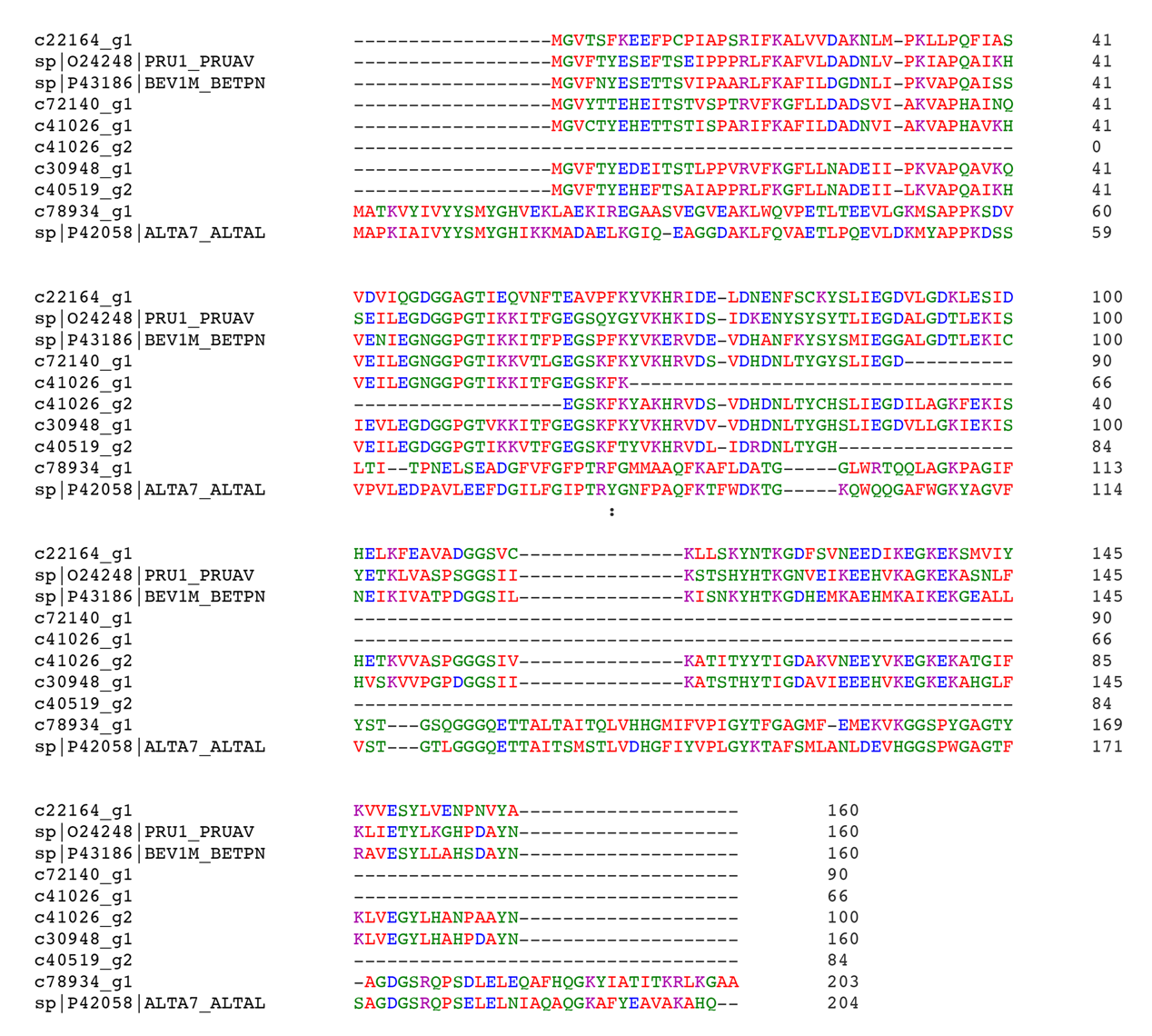

Supplement: Supplementary file 10 — Figure S7. Protein sequence alignment of allergenic proteins and the identified homologs from fig. PRU1_PRUAV: major allergen Pru av. 1 of Prunus avium; BEV1M_BETPN: major pollen allergen Bet v 1-M/N of Betula pendula; ALTA7_ALTAL: minor allergen Alt a 7 of Alternaria alternata. Two dots, similar positions. (DOCX 4251 kb) [file 12870_2019_1742_MOESM10_ESM.docx]
